# Intracranial and subcortical volumes in adolescents with early‐onset psychosis: A multisite mega‐analysis from the ENIGMA consortium

**DOI:** 10.1002/hbm.25212

**Published:** 2020-10-05

**Authors:** Tiril P. Gurholt, Vera Lonning, Stener Nerland, Kjetil N. Jørgensen, Unn K. Haukvik, Clara Alloza, Celso Arango, Claudia Barth, Carrie E. Bearden, Michael Berk, Hannes Bohman, Orwa Dandash, Covadonga M. Díaz‐Caneja, Carl T. Edbom, Theo G. M. van Erp, Anne‐Kathrin J. Fett, Sophia Frangou, Benjamin I. Goldstein, Anahit Grigorian, Neda Jahanshad, Anthony C. James, Joost Janssen, Cecilie Johannessen, Katherine H. Karlsgodt, Matthew J. Kempton, Peter Kochunov, Lydia Krabbendam, Marinos Kyriakopoulos, Mathias Lundberg, Bradley J. MacIntosh, Bjørn Rishovd Rund, Runar E. Smelror, Alysha Sultan, Christian K. Tamnes, Sophia I. Thomopoulos, Ariana Vajdi, Kirsten Wedervang‐Resell, Anne M. Myhre, Ole A. Andreassen, Paul M. Thompson, Ingrid Agartz, For the ENIGMA‐EOP Working Group

**Affiliations:** ^1^ Norwegian Center for Mental Disorders Research (NORMENT), Division of Mental Health and Addiction Oslo University Hospital Oslo Norway; ^2^ Norwegian Center for Mental Disorders Research (NORMENT), Institute of Clinical Medicine University of Oslo Oslo Norway; ^3^ Department of Psychiatric Research Diakonhjemmet Hospital Oslo Norway; ^4^ Department of Adult Mental Health, Institute of Clinical Medicine University of Oslo Oslo Norway; ^5^ Department of Child and Adolescent Psychiatry, Institute of Psychiatry and Mental Health Hospital General Universitario Gregorio Marañón, IiSGM, CIBERSAM Madrid Spain; ^6^ School of Medicine Universidad Complutense Madrid Spain; ^7^ Department of Psychiatry and Biobehavioral Sciences, Semel Institute for Neuroscience and Human Behavior UCLA Los Angeles California USA; ^8^ Department of Psychology UCLA Los Angeles California USA; ^9^ IMPACT, The Institute for Mental and Physical Health and Clinical Translation, School of Medicine, Deakin University Geelong Victoria Australia; ^10^ Orygen Youth Health Research Center The Florey Institute for Neuroscience and Department of Psychiatry Parkville Victoria Australia; ^11^ Center for Psychiatry Research, Department of Clinical Neuroscience Karolinska Institutet and Stockholm Health Care Services, Stockholm Region, Stockholm, Sweden Stockholm Sweden; ^12^ Department of Neuroscience, Child and Adolescent Psychiatry Uppsala University Uppsala Sweden; ^13^ Department of Clinical Science and Education Södersjukhuset Karolinska Institutet Stockholm Sweden; ^14^ Clinical Translational Neuroscience Laboratory, Department of Psychiatry and Human Behavior University of California Irvine Irvine California USA; ^15^ Center for the Neurobiology of Learning University of California Irvine and Memory Irvine California USA; ^16^ Department of Psychology City, University of London London UK; ^17^ Department of Psychosis Studies IoPPN London UK; ^18^ Department of Clinical, Neuro and Developmental Psychology VU Amsterdam Amsterdam Netherlands; ^19^ Department of Psychiatry Icahn School of Medicine at Mount Sinai New York New York USA; ^20^ Center for Youth Bipolar Disorder, Sunnybrook Health Science Center Toronto Ontario Canada; ^21^ Department of Psychiatry and Pharmacology University of Toronto Canada; ^22^ Imaging Genetics Center, Mark and Mary Stevens Neuroimaging and Informatics Institute, Keck School of Medicine University of Southern California Marina del Rey California USA; ^23^ Department of Psychiatry University of Oxford Oxford UK; ^24^ Oxford Health Foundation NHS Trust Oxford UK; ^25^ Department Psychiatry and Biobehavioral Sciences UCLA Los Angeles California USA; ^26^ Department of Psychosis Studies King's College London London UK; ^27^ Maryland Psychiatric Research Center, Department of Psychiatry University of Maryland School of Medicine Baltimore Maryland USA; ^28^ Department of Child and Adolescent Psychiatry, Institute of Psychiatry, Psychology, and Neuroscience King's College London London UK; ^29^ National and Specialist Children's Inpatient Unit (Acorn Lodge), South London and Maudsley NHS Foundation Trust Beckenham UK; ^30^ The Department of Clinical Science and Education KI SÖS Stockholm Sweden; ^31^ Hurvitz Brain Sciences, Sunnybrook Research Institute Toronto Ontario Canada; ^32^ Department of Medical Biophysics University of Toronto Ontario Canada; ^33^ Department of Psychology University of Oslo Oslo Norway; ^34^ Department of Research Vestre Viken Hospital Trust Drammen Norway; ^35^ Department of Pharmacology University of Toronto Toronto Ontario Canada; ^36^ PROMENTA Research Center, Department of Psychology University of Oslo Oslo Norway; ^37^ Child and Adolescent Psychiatry Unit, Division of Mental Health and Addiction, Institute of Clinical Medicine University of Oslo Oslo Norway; ^38^ Department of Psychiatric Research and Development, Division of Mental Health and Addiction Oslo University Hospital Oslo Norway

**Keywords:** adolescence, antipsychotics, brain structure, early‐onset, intracranial volume, psychosis spectrum

## Abstract

Early‐onset psychosis disorders are serious mental disorders arising before the age of 18 years. Here, we investigate the largest neuroimaging dataset, to date, of patients with early‐onset psychosis and healthy controls for differences in intracranial and subcortical brain volumes. The sample included 263 patients with early‐onset psychosis (mean age: 16.4 ± 1.4 years, mean illness duration: 1.5 ± 1.4 years, 39.2% female) and 359 healthy controls (mean age: 15.9 ± 1.7 years, 45.4% female) with magnetic resonance imaging data, pooled from 11 clinical cohorts. Patients were diagnosed with early‐onset schizophrenia (*n* = 183), affective psychosis (*n* = 39), or other psychotic disorders (*n* = 41). We used linear mixed‐effects models to investigate differences in intracranial and subcortical volumes across the patient sample, diagnostic subgroup and antipsychotic medication, relative to controls. We observed significantly lower intracranial (Cohen's *d* = −0.39) and hippocampal (*d* = −0.25) volumes, and higher caudate (*d* = 0.25) and pallidum (*d* = 0.24) volumes in patients relative to controls. Intracranial volume was lower in both early‐onset schizophrenia (*d* = −0.34) and affective psychosis (*d* = −0.42), and early‐onset schizophrenia showed lower hippocampal (*d* = −0.24) and higher pallidum (*d* = 0.29) volumes. Patients who were currently treated with antipsychotic medication (*n* = 193) had significantly lower intracranial volume (*d* = −0.42). The findings demonstrate a similar pattern of brain alterations in early‐onset psychosis as previously reported in adult psychosis, but with notably low intracranial volume. The low intracranial volume suggests disrupted neurodevelopment in adolescent early‐onset psychosis.

## Graphical abstract

Early‐onset psychosis disorders are serious mental disorders, arising before the age of 18 years, and affect 0.05% to 0.5% of the population. In the largest neuroimaging study, to date, of patients with early‐onset psychosis and healthy controls, we investigate for differences in intracranial and subcortical brain volumes. The findings demonstrate a similar brain alteration in early‐onset psychosis as previously reported for adult psychosis, but with a notably low intracranial volume, which may suggest greater neurodevelopmental mechanisms in early‐onset psychosis.

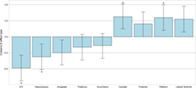

## 1 INTRODUCTION

Early‐onset psychosis (EOP) disorders, defined as psychotic disorders with onset of the first psychotic episode before age 18 years, affect 0.05–0.5% of the population (Boeing et al., 2007; Dalsgaard et al., 2019; Gillberg, Wahlström, Forsman, Hellgren, & Gillberg, 1986; Sikich, 2013), and have a heterogeneous clinical presentation. They are debilitating mental disorders and constitute one of the leading causes of lifetime disease burden for adolescents (Gore et al., 2011). With the exception of some prior multisite studies (Arango et al., 2012; Fraguas, Díaz‐Caneja, Pina‐Camacho, Janssen, & Arango, 2016; Reig et al., 2011), structural brain magnetic resonance imaging (MRI) studies in EOP are limited by small sample sizes and low statistical power, typically including fewer than 50 patients. To overcome these limitations, the Enhancing Neuro Imaging Genetics through Meta Analysis (ENIGMA; http://enigma.ini.usc.edu) Early‐onset Psychosis Working Group was established, and this first ENIGMA‐EOP study of intracranial and subcortical brain volumes includes data from 11 participating sites.

Prior studies of EOP have shown lower overall brain volume (El‐Sayed, 2010; Frazier et al., 1996a; Matsumoto et al., 2001), alterations in cortical gray matter volume/thickness (Arango et al., 2012; Janssen et al., 2012; Janssen et al., 2014; Reig et al., 2011; Thormodsen et al., 2013), lower thalamus (Frazier et al., 1996a; Janssen et al., 2012), and higher caudate, putamen, pallidum (Frazier et al., 1996a) and ventricular volumes (Frazier, Giedd, Hamburger, et al., 1996; Juuhl‐Langseth et al., 2012; Pagsberg et al., 2007; Sowell et al., 2000), compared to healthy controls. Large‐scale ENIGMA meta‐analyses in adult schizophrenia (van Erp et al., 2016) and bipolar disorder (Hibar et al., 2016) reported lower hippocampal, amygdala and thalamus, and higher lateral ventricle volumes; and additionally lower intracranial (ICV) and accumbens volumes, and higher pallidum volume in schizophrenia (van Erp et al., 2016). These volumetric differences were less marked in bipolar disorder than in schizophrenia, a finding corroborated by a direct comparison study (Rimol et al., 2010). In teenagers with childhood‐onset schizophrenia, one study have indicated similar magnitudes of subcortical volume differences to that of adult schizophrenia (Frazier et al., 1996a). Yet, it is still not clear how similar brain structural alterations in EOP are to those of adult‐onset psychosis, or to other neurodevelopmental disorders such as autism spectrum disorder (ASD) and attention deficit hyperactivity disorder (ADHD).

Only two EOP studies have investigated a comprehensive array of subcortical structures (Frazier et al., 1996a; Juuhl‐Langseth et al., 2012). These reported higher caudate, lateral and fourth ventricular volumes (Juuhl‐Langseth et al., 2012), and higher caudate, putamen and pallidum volumes that correlated with neuroleptic treatment and age of onset (Frazier et al., 1996a). A study on early‐onset bipolar disorder, where 61% of patients had psychosis symptoms, reported lower amygdala and higher putamen volumes (DelBello, Zimmerman, Mills, Getz, & Strakowski, 2004). It has been difficult to establish a consistent pattern of subcortical alterations in EOP from these studies, due to the limited number of previous studies and their relatively low sample sizes.

Antipsychotic treatment has been linked to basal ganglia volume increases in adult patients (Chakos et al., 1994; Chua et al., 2009; di Sero et al., 2019; Hashimoto et al., 2018; van Erp et al., 2016). Although not consistently replicated (Emsley et al., 2015), this finding may reflect striatal hypertrophy as a compensatory response to striatal dopamine receptor antagonism (Chua et al., 2009), the main target of antipsychotics, and a link between antipsychotic dose and basal ganglia volume has been shown (Andersen et al., 2020; di Sero et al., 2019). This proposed mechanism is likely mediated by the dopamine D2 receptor (Guma et al., 2018), and the neurobiological responses may differ as a function of neurodevelopmental phase (Moe, Medely, Reeks, Burne, & Eyles, 2017). In patients with childhood‐onset schizophrenia (i.e., onset before age 13 years) investigated during adolescence, pallidum volumes enlarged with neuroleptic exposure (Frazier et al., 1996a) while transfer to clozapine treatment was associated with caudate volume reduction (Frazier et al., 1996b). In early‐onset schizophrenia (i.e., onset before age 18 years), antipsychotic medication use was associated with ventricular enlargement but not with higher caudate volumes (Juuhl‐Langseth et al., 2012).

The aim of this study was to robustly identify differences in ICV and subcortical volumes using a mega‐analytical approach^1^ of the hitherto largest neuroimaging sample of adolescent patients with EOP and healthy controls. We performed subgroup analyses by dividing patients into three groups: (a) early‐onset schizophrenia, (b) affective psychosis disorders, including bipolar and major depressive disorders with psychotic features, and (c) other psychoses, including psychosis not otherwise specified, and brief psychotic disorders. Based on prior adult studies (Hibar et al., 2016; Rimol et al., 2010; van Erp et al., 2016), we hypothesized lower ICV and volumetric subcortical abnormalities in EOP similar to those of adult psychosis when compared to healthy controls; with strongest effects in early‐onset schizophrenia, and an attenuated but similar pattern in affective psychosis and other psychoses. Based on prior studies on antipsychotic treatment (Chakos et al., 1994; Chua et al., 2009; di Sero et al., 2019; Frazier et al., 1996a; Hashimoto et al., 2018; van Erp et al., 2016), we expected antipsychotic medication use in EOP to be associated with enlarged basal ganglia volumes.

## 2 MATERIALS AND METHODS

### 2.1 Study samples

Eleven cohorts with MRI data acquired on 16 scanners (1.5T: four scanners/223 scans; 3T: 12 scanners/399 scans) contributed to this study, yielding a combined sample of 263 patients with EOP and 359 healthy controls. The patient group included early‐onset schizophrenia (*n* = 183), affective psychosis (*n* = 39), or other psychosis (*n* = 41) according to DSM‐IV (American Psychiatric Association, 2000) or ICD‐10 (World Health Organization, 2004). Patients were between age 12 to 18 years at scan (14 had illness onset before age 12). Table 1 presents demographic and clinical information for the combined sample. Table S1 demonstrates cohort‐wise demographic and clinical information, and Table S2 cohort‐specific inclusion and exclusion criteria. Five sites shared positive and negative syndrome (PANSS; Kay, Fiszbein, & Opler, 1987) scores, while one site shared Scale for the Assessment of Negative/Positive Symptoms (SANS [Andreasen, 1983]/SAPS [Andreasen, 1984]) scores.

**TABLE 1:** Demographic and clinical information

	Patients (*N* = 263)	Controls (*N* = 359)	χ^2^‐test/Wilcoxon rank‐sum test	*p* value
	*N* (%)	*N* (%)		
Females	103 (39.2%)	163 (45.4%)	2.2	.1410

	Mean ± *SD*	Mean ± *SD*		
Age^a^ (years)	16.4 ± 1.4	15.9 ± 1.7	0.5	.0003
Age at onset^b^ (years)	15.0 ± 1.9			
Duration of illness^b^ (years)	1.5 ± 1.4			
PANSS positive^c^	18.9 ± 7.7			
PANSS negative^c^	18.1 ± 7.6			

	Range (median)	Range (median)		
Age (years)	12–18.9 (16.7)	12–18.9 (16)		
Age of onset^b^ (years)	7.6–18.2 (15.2)			
Duration of illness^b^ (years)	0–7.5 (1.0)			
PANSS positive^c^	7–46 (19)			
PANSS negative^c^	7–43 (16)			
	*N*	*N*		
EOS/AFP/OTP	183/39/41			
AP users/non‐users^d^	193/39			
AP users EOS/AFP/OTP^d^	136/26/31			
1.5T/3T	117/146	106/253		

*Note: p* values < .05 considered significant.

Abbreviations: AFP, affective psychosis; AP, antipsychotics; EOS, early‐onset schizophrenia; OTP, other psychoses; PANSS, positive and negative syndrome scale.

a Not normally distributed, applied Wilcoxon rank‐sum test.

b 32 had missing AAO/DOI.

c 90 had missing PANSS positive/negative scores.

d 31 (30 EOS, one AFP) had missing information on current AP medication.

All participants, parents or legal guardians gave written informed consent or assent as appropriate. Each site had ethical approval from their local ethics committees or institutional review board to participate in the study and share anonymized data for mega‐analysis. The study was conducted in accordance with the Helsinki Declaration.

### 2.2 MR imaging acquisition and processing

T1‐weighted brain images were processed locally at each site with FreeSurfer (version 5.3.0; http://surfer.nmr.mgh.harvard.edu; Fischl, 2012) following standardized ENIGMA processing protocols (http://enigma.ini.usc.edu). See Table S3 for cohort/scanner‐specific acquisition parameters. In the statistical analyses, we included the same brain measures as prior ENIGMA studies (Hibar et al., 2016; van Erp et al., 2016), namely estimated ICV (Buckner et al., 2004) and bilateral subcortical volumes: accumbens, amygdala, hippocampal, thalamus, lateral ventricle, caudate, pallidum, and putamen.

The segmentation quality was assessed following a standardized protocol by identifying outlier volumes at each site, which were excluded if the segmentation was deemed inaccurate after visual inspection (Note S1).

### 2.3 Statistical analyses

The demographic variables of patients and controls were compared using χ^2^‐test for categorical variables and *t*‐test/two‐sided Wilcoxon rank sum test for normally/non‐normally distributed continuous variables. Normality was assessed using the Shapiro–Wilk test. We evaluated the distribution of the participants age (Figure S1) and brain volumes (Figure S2).

We investigated ICV and subcortical volumes mega‐analytically with linear‐mixed effects (LME) models (Laird & Ware, 1982) using the *lme* function (*nlme* package) in R (version 3.5.2; www.r-project.org). We adjusted for age, sex, and ICV (subcortical volumes only) as fixed‐effects variables unless otherwise specified, and scanner as random‐effects variable. Independent analyses were conducted for combined (averaged), and left/right hemispheric structures. We refer to combined structures unless otherwise specified.

In the *main model*, we investigated patient‐control differences in ICV and subcortical volumes. For qualitative comparison of between‐cohort heterogeneity, we included a complementary meta‐analysis (Note S2). Follow‐up analyses were performed for age‐by‐group or sex‐by‐group interactions. Additionally, we conducted follow‐up analyses of the main model without adjusting for ICV to explore the relative difference in subcortical structures.

Next, we conducted follow‐up analyses of the main LME model for patient characteristics on brain volumes. We stratified patients into early‐onset schizophrenia, affective psychosis, and other psychoses, and compared each diagnostic subgroup to controls. Similarly, we stratified patients into current antipsychotic users and current non‐users, and compared them to controls. Finally, in patients, we tested for effects of age at onset, duration of illness, and antipsychotic medication users versus non‐users, on brain volumes. These analyses were adjusted for age, sex, and ICV as fixed‐effects variables and scanner as random‐effects variable.

We computed the Cohen's *d* effect sizes from the *t*‐statistics for categorical variables and via the partial correlation coefficient (*r*) for continuous variables (Nakagawa & Cuthill, 2007). We corrected for multiple comparisons using the false discovery rate (FDR; Benjamini & Hochberg, 1995) with *α* = 0.05 and computed a study‐wide FDR threshold across *N* = (9 + 2*8)*(8 + 2) + (9 + 8*2)*3 = 325 tests, which is the number of reported combined, left‐ and right‐hemisphere tests, leading us to consider *p* values ≤.0062 as significant. Nominally significant *p* values (*p* ≤ .05) are reported. We report uncorrected *p* values. Tables S4–S15 present the results of the individual analyses.

## 3 RESULTS

### 3.1 Demographics and clinical information

Patients with EOP were significantly older than controls (Δ = 0.5, *p* = 3 × 10^−4^). The largest patient group was early‐onset schizophrenia (69.5%), followed by other psychoses (15.6%), and affective psychosis (14.8%). The patients had mean illness duration 1.5 ± 1.4 years, age at onset 15.0 ± 1.9 years, and PANSS positive and negative scores of 18.9 ± 7.7 and 18.1 ± 7.6, respectively. 193 (73.4%) patients received current treatment with antipsychotic medication (Table 1). Split by diagnosis, 136 (74.3%) early‐onset schizophrenia, 31 (75.6%) other psychosis, and 26 (66.7%) affective psychosis patients currently used antipsychotic medication.

### 3.2 Case–control differences

**FIGURE 1:**
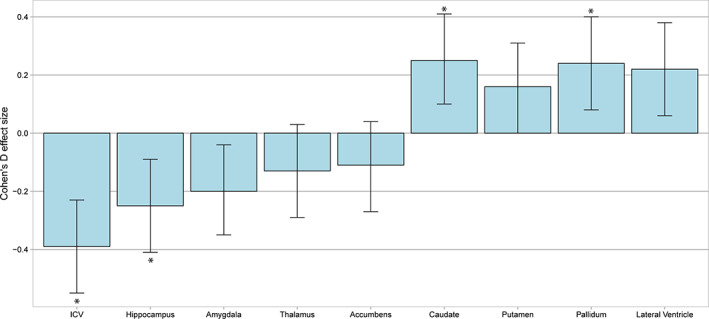
Regional brain volumes in early‐onset psychosis compared to healthy controls. Notes: Linear mixed‐effects models applied for diagnostic differences between patients with early‐onset psychosis and healthy controls (reference), adjusted for age, sex, and ICV (for subcortical structures) as fixed‐effects variables and scanner as a random‐effects variable. Error bars show pooled effect size ± standard error. Significant differences indicated by *

EOP had significantly lower ICV (Cohen's *d* = −0.39, *p* = 3 × 10^−6^) and hippocampal volume (*d* = −0.25, *p* = .003), higher caudate (*d* = 0.25, *p* = .002), and pallidum (*d* = 0.24, *p* = .004) volumes, and nominally significant lower amygdala (*d* = −0.2, *p* = .020), and higher lateral ventricular (*d* = 0.22, *p* = .010) volumes, compared to controls (Figure 1).

In split‐hemisphere analyses, only structures of the left hemisphere were significantly altered, showing lower hippocampal (*d* = −0.24, *p* = .005), and higher caudate (*d* = 0.27, *p* = .001), pallidum (*d* = 0.28, *p* = 8e‐04), and lateral ventricular (*d* = 0.24, *p* = .004) volumes (Table S4).

Follow‐up analyses did not show significant age‐by‐group or sex‐by‐group interactions (Tables S5 and S6).

In follow‐up analyses unadjusted for ICV, we observed significantly lower hippocampal (*d* = −0.42, *p* = 6 × 10^−7^), amygdala (*d* = −0.32, *p* = 2 × 10^−4^), thalamus (*d* = −0.32, *p* = 1 × 10^−4^), and accumbens (*d* = −0.25, *p* = .004) volumes in EOP relative to controls (Figure S3). Effects were similar bilaterally (Table S7).

The complementary meta‐analysis of patient‐control differences largely corroborated the results, but additionally showed significantly higher lateral ventricular volume (*d* = 0.3, *p* = 5 × 10^−4^), with similar effects bilaterally (Table S8). Forest plots illustrate the variability among cohorts (Figures S4–S6).

### 3.3 Diagnostic subgroups

**FIGURE 2:**
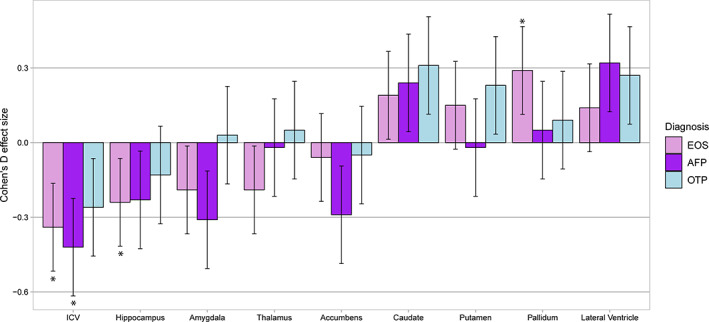
Regional brain volumes in early‐onset psychosis patient subgroups compared to healthy controls. Notes: Linear mixed‐effects models applied for diagnostic differences in patient subgroups (EOS/AFP/OTP) with controls (reference), adjusted for age, sex, and ICV (for subcortical structures) as fixed‐effects variables and scanner as a random‐effects variable. Error bars show mean effect size ± standard error. Significant differences indicated by *. AFP, affective psychosis; EOS, early‐onset schizophrenia; OTP, other psychoses

When stratifying by EOP diagnostic subgroups, both early‐onset schizophrenia (*d* = −0.34, *p* = 1 × 10^−4^) and affective psychosis (*d* = −0.42, *p* = .003) showed lower ICV, while only early‐onset schizophrenia showed significantly lower hippocampal (*d* = −0.24, *p* = .006) and higher pallidum (*d* = 0.29, *p* = 9 × 10^−4^) volumes relative to controls. There were nominally significant differences for amygdala (schizophrenia: *d* = −0.19, *p* = .029; affective psychosis: *d* = −0.31, *p* = .026), thalamus (schizophrenia: *d* = −0.19, *p* = .031), accumbens (affective psychosis: *d* = −0.29, *p* = .041), caudate (schizophrenia: *d* = 0.19, *p* = .029; other psychosis: *d* = 0.31, *p* = .024), and lateral ventricle (affective psychosis: *d* = 0.32, *p* = .024; other psychosis: *d* = 0.27, *p* = .048; Figure 2, Table S9) volumes. In split‐hemisphere analyses, early‐onset schizophrenia showed significantly lower hippocampal (left: *d* = −0.23, *p* = .008) and higher pallidum (left: *d* = 0.28, *p* = .001; right: *d* = 0.24, *p* = .006) volumes, while affective psychosis showed lower left accumbens (*d* = −0.39, *p* = .005) volume (Tables S10 and S11).

### 3.4 Age at onset and illness duration

There were no significant effects of age at onset or duration of illness on the included regional brain volumes in EOP (Tables S12 and S13).

### 3.5 Antipsychotic medication

**FIGURE 3:**
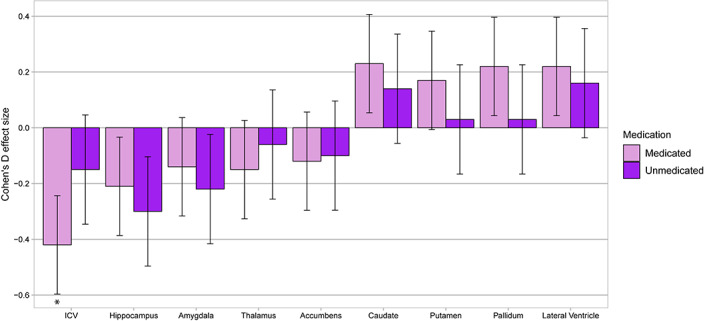
Regional brain volumes by current antipsychotic medication status in early‐onset psychosis compared to healthy controls. Notes: Linear mixed‐effects models investigating the effect of antipsychotic medication user/non‐user with controls (reference), adjusted for age, sex, and ICV (for subcortical structures) as fixed‐effects variables and scanner as a random‐effects variable. Error bars show mean effect size ± standard error. Significant differences indicated by *

When stratifying patients based on current antipsychotic medication use, users (*n* = 193) had significantly lower ICV (*d* = −0.42, *p* = 2 × 10^−6^), and nominally significant lower hippocampal, and higher caudate, pallidum, and lateral ventricular volumes relative to controls (Figure 3, Table S14). In split‐hemisphere analyses, antipsychotic users showed significantly higher left caudate (*d* = 0.24, *p* = .006) and pallidum (*d* = 0.26, *p* = .003) volumes compared to controls. Non‐users (*n* = 39) showed nominally significant lower hippocampal volume.

Direct comparison between antipsychotic users/non‐users showed no significant volumetric differences (Table S15).

## 4 DISCUSSION

In this mega‐analysis, we found a structural brain signature characterized by significantly lower ICV and hippocampal volumes, and higher caudate and pallidum volumes in EOP compared to healthy controls. Lower ICV featured in both early‐onset schizophrenia and affective psychosis, while lower hippocampal and higher pallidum volumes were limited to schizophrenia. Enlarged left caudate and pallidum volumes were observed in patients currently on antipsychotic treatment.

**FIGURE 4:**
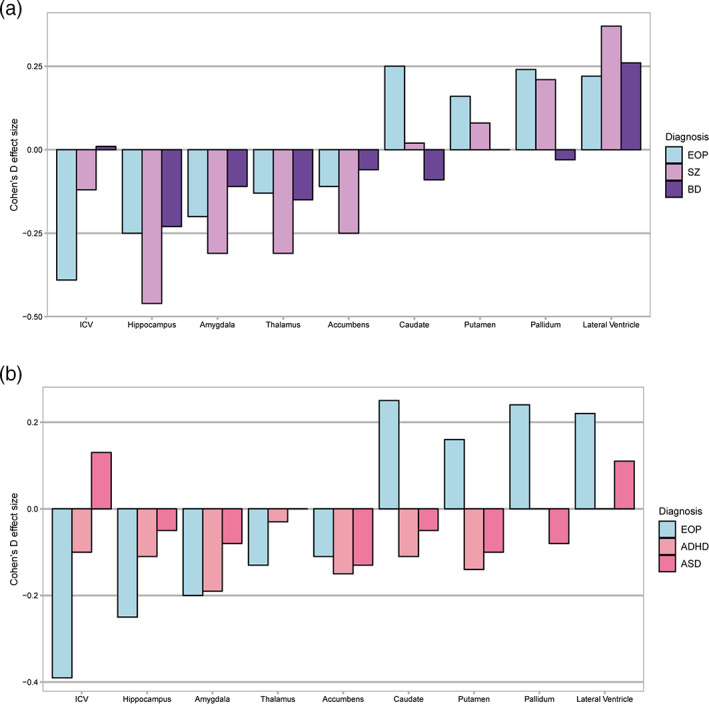
Effect sizes observed in early‐onset psychosis compared to other neurodevelopmental disorders from prior ENIGMA publications. Notes: Figure compares the effect sizes that we observe in EOP with those from prior ENIGMA publications; In (a) meta‐analysis of adult schizophrenia (van Erp et al., 2016) and bipolar disorder (Hibar et al., 2016), and in (b) mega‐analyses of life‐span ADHD (Hoogman et al., 2017) and ASD (van Rooij et al., 2018). The age‐ranges and healthy control populations differs among the studies, and the ADHD study did not report on the lateral ventricles. ADHD, attention deficit hyperactivity disorder; ASD, autism spectrum disorder; BD, bipolar disorder; EOP, early‐onset psychosis; SZ, schizophrenia

The most striking finding was the lower ICV in patients with EOP (*d* = −0.39). This effect was larger than the lower ICV reported in a meta‐analysis of adult schizophrenia (*d* = −0.12; van Erp et al., 2016), and no such effect was reported for adult bipolar disorders (Hibar et al., 2016) (Figure 4a). ICV expands rapidly in early age, driven by brain growth, and is thought to reach 90% of its final volume by early adolescence (Courchesne et al., 2000; Haijma et al., 2013). A study combining multiple longitudinal samples found evidence for continued ICV expansion, with an estimated annual increase of ~1% from late childhood to mid‐adolescence, with stabilization in late adolescence (Mills et al., 2016). Given the relative stability of ICV from mid‐adolescence onwards, our findings of lower ICV in EOP suggest an aberrant neurodevelopment, which is more severe and/or established earlier than in adult‐onset psychosis.

Interestingly, a prior multisite study found lower ICV in ADHD (*d* = −0.10), with strongest effects among children (*d* = −0.14) and adolescents (*d* = −0.13; not significant; Hoogman et al., 2017), which is smaller than the effect size observed in EOP (*d* = −0.39; Figure 4b). In contrast, higher ICV was reported in ASD (*d* = 0.13; van Rooij et al., 2018), indicating differential neurodevelopmental mechanisms. Our findings further highlight the importance of considering ICV in imaging studies of neurodevelopmental disorders, and suggest the need for research into early mechanisms influencing smaller head development, including longitudinal studies and normative modeling (Wolfers et al., 2018) of growth patterns from birth, and their relationship to cognitive development and functioning.

The subcortical signature of EOP appeared similar to that of adult schizophrenia (van Erp et al., 2016) and bipolar disorder (Hibar et al., 2016) relative to controls (Figure 4a). Similarities included lower hippocampal, and higher pallidum (schizophrenia only) and lateral ventricular (nominally significant in EOP) volumes; except for pallidum, effect sizes were less pronounced than in schizophrenia. Additionally, we confirmed the higher caudate volumes, as previously observed in EOP (Frazier et al., 1996b; Juuhl‐Langseth et al., 2012). Generally, the magnitude of subcortical differences in EOP relative to controls appeared greater than those reported in the ADHD (Hoogman et al., 2017) or ASD (van Rooij et al., 2018) samples, and the directionality of effects were similar for limbic structures, while opposing for basal ganglia structures (Figure 4b).

The hippocampus showed the lowest volume in EOP relative to controls, in agreement with findings in adult schizophrenia (van Erp et al., 2016) and bipolar disorder (Hibar et al., 2016). The effect size (*d* = −0.25) was similar to those reported in bipolar disorder (*d* = −0.23; Hibar et al., 2016), but smaller than in schizophrenia (*d* = −0.46; van Erp et al., 2016). Lower hippocampal volume was also reported in children (*d* = −0.12; <15 years) and adolescents (*d* = −0.24; age‐range 15–21 years) with ADHD (Hoogman et al., 2017). Thus, lower hippocampal volume features across several neurodevelopmental disorders, and may reflect both shared and distinct illness mechanisms. Adult studies have shown hippocampal subfield alterations in schizophrenia and bipolar disorder (Haukvik et al., *submitted* 2020; Haukvik, Tamnes, Söderman, & Agartz, 2018). Future EOP studies should track volumetric change across the illness course and perform fine‐scale mapping to advance our understanding of both the timing and location of hippocampal disruption in neurodevelopmental disorders.

Our finding of lateral ventricular enlargement in EOP (nominally significant combined structure, significant left hemisphere) corresponded closely with the adult literature, but showed a smaller effect size than reported for both adult schizophrenia (van Erp et al., 2016) and bipolar disorder (Hibar et al., 2016). Prior studies indicate progressive ventricular expansion across the illness course (Kempton, Stahl, Williams, & DeLisi, 2010). Thus, the short illness duration of patients with EOP could explain the smaller effect size, but longitudinal studies are needed to examine this possibility.

In contrast to studies on adult schizophrenia (van Erp et al., 2016), we did not detect significantly lower amygdala, thalamus and accumbens, or higher putamen volumes. However, we observed trends in the same direction and therefore cannot rule out that the smaller sample size or short duration of illness could explain some of these differences. Further, when omitting ICV as a fixed‐effects variable, these volumes (except putamen) were significantly lower than in controls. Since EOP shows larger effect sizes for ICV than for adult schizophrenia (van Erp et al., 2016), adjusting for ICV may partly explain the difference in results. This suggests that the relative difference for amygdala, thalamus and accumbens volumes is larger in EOP versus adolescent controls, than for adult schizophrenia.

In the subgroup analyses, we observed lower ICV in both early‐onset schizophrenia and affective psychosis. Significantly lower hippocampal and higher pallidum volumes were limited to early‐onset schizophrenia, while lower left accumbens was observed in affective psychosis. Since most patients had a diagnosis of early‐onset schizophrenia, the subgroup differences may be explained by the unbalanced sizes of the subgroups. Indeed, comparable effect sizes were observed for hippocampal volume in early‐onset schizophrenia (*d* = −0.24; *n* = 183) and affective psychosis (*d* = −0.23; *n* = 39); although not significant in affective psychosis. The supplementary meta‐analysis suggests a great degree of heterogeneity across the included samples, which could reflect differences in inclusion and exclusion procedures and criteria, or subgroup differences, resulting in variable representation. This further highlights the necessity for large samples when characterizing heterogeneous disorders composed of clinically diverse subgroups.

Our findings of significantly enlarged left pallidum and caudate volumes, and trends towards enlarged putamen volumes in antipsychotic medicated patients suggest similar medication effects on brain structure in adolescents and adults. Intriguingly, the significantly higher caudate volume seen in EOP was not observed in adult schizophrenia (van Erp et al., 2016) or bipolar disorder (Hibar et al., 2016). In past meta‐analyses of schizophrenia (Haijma et al., 2013; Keshavan et al., 1994), lower caudate volumes were seen in antipsychotic‐naive patients but not in medicated patients. We did not find lower caudate volume in non‐medicated patients compared to controls, which may point to a relative conservation of caudate volume in early‐onset compared to adult‐onset psychosis. However, the proportion of patients not using antipsychotics was low; therefore, the comparisons between users and non‐users may have been underpowered. Furthermore, we could not ascertain that the non‐medicated patients were naïve to previous antipsychotic medication. In a previous study of early‐onset schizophrenia, the enlarged caudate volumes were not linked to antipsychotic medication use (Juuhl‐Langseth et al., 2012), while in adolescents with childhood‐onset schizophrenia who initially were on typical neuroleptics, caudate enlargement declined following clozapine treatment (Frazier et al., 1996b).

This study had some limitations. The cross‐sectional design precluded the investigation of developmental trajectories. Data was collected from different sites, and site‐wise differences in diagnostic routines and MRI acquisition may influence the results. ICV and height show genetic correlation (Adams et al., 2016). We did not have information on height/weight, and could not test whether small body size explained the ICV differences. The validity of the FreeSurfer estimated ICV has been questioned (Heinen et al., 2016; Klasson, Olsson, Eckerström, Malmgren, & Wallin, 2018), and it is an indirect measurement of the intracranial volume. In the medication analysis, we did not adjust for patient subgroup or illness severity that may influence the results. We could not ascertain possible prior antipsychotic use in current non‐users, nor putative effects of cumulative antipsychotic exposure, antipsychotic type and dosage, age at initial treatment, or the effects of lithium and other mood stabilizers.

Strengths of this study include the hitherto largest adolescent EOP sample, harmonized and validated ENIGMA MRI processing protocols, and a mega‐analytical approach previously shown to be more sensitive than meta‐analysis (Boedhoe et al., 2019). The standardized analysis pipeline enabled comparative discussion with results from prior ENIGMA studies. Patients with EOP likely have shorter illness duration, less treatment exposure, shorter history of adverse lifestyle, and less alcohol/substance use, compared to adult patients. Thus, the influence of these confounding variables should be comparably smaller than in adult samples.

## 5 CONCLUSION

The pattern of volumetric differences in EOP overlapped with, but also showed notable deviations from findings as previously demonstrated in adult psychosis studies. Particularly, the large effect size for ICV may point to a greater involvement of neurodevelopmental mechanisms in EOP, possibly serving as a predictor for early psychosis risk. Future studies should investigate the biological mechanisms of low ICV to identify potential risk markers and targets for differential psychiatric treatment in EOP. This study illustrates the benefits of large‐scale international collaborative efforts in characterizing brain structural abnormalities in rare disorders and lays the groundwork for future studies of the neural foundations for EOP.

## CONFLICT OF INTEREST

Celso Arango: has been a consultant to or has received honoraria or grants from Acadia, Angelini, Gedeon Richter, Janssen Cilag, Lundbeck, Otsuka, Roche, Sage, Servier, Shire, Schering Plough, Sumitomo Dainippon Pharma, Sunovion, and Takeda; Covadonga M. Díaz‐Caneja: has received honoraria from AbbVie, Sanofi, and Exeltis; Michael Berk: was supported by an unrestricted grant from AstraZeneca; Paul M. Thompson, Neda Jahanshad: MPI of a research grant from Biogen, Inc., for work unrelated to the contents of this manuscript. Ole A. Andreassen: has received speaker's honorarium from Lundbeck, and is a consultant to HealthLytix. Bradley J. MacIntosh: received a NARSAD Independent Investigator award from the Brain Behavior Research Foundation.

## AUTHOR CONTRIBUTIONS

Cohort PI: Celso Arango, Carrie E. Bearden, Michael Berk, Anne‐Kathrin J. Fett, Lydia Krabbendam, Sophia Frangou, Benjamin I. Goldstein, Anthony C. James, Katherine H. Karlsgodt, Marinos Kyriakopoulos, Mathias Lundberg, Bjørn R. Rund, Anne M. Myhre, Ingrid Agartz. Project development: Tiril P. Gurholt, Vera Lonning, Stener Nerland, Unn K. Haukvik, Carl T. Edbom, Anne‐Kathrin J. Fett, Marinos Kyriakopoulos, Neda Jahanshad, Sophia I. Thomopoulos, Paul M. Thompson, Ingrid Agartz. Data organization/handling: Tiril P. Gurholt. Statistical analysis: Tiril P. Gurholt, Stener Nerland, Vera Lonning. Data interpretation: Tiril P. Gurholt, Vera Lonning, Stener Nerland, Kjetil N. Jørgensen, Carrie E. Bearden, Bradley J. MacIntosh. Preparation of results/illustrations: Tiril P. Gurholt. Manuscript preparation: Tiril P. Gurholt, Vera Lonning, Stener Nerland, Unn K. Haukvik, Kjetil N. Jørgensen, Carrie E. Bearden, Christian K. Tamnes, Hannes Bohman, Claudia Barth, Paul M. Thompson, Ole A. Andreassen, Ingrid Agartz. Imaging Data collection: Stener Nerland, Clara Alloza, Carrie E. Bearden, Michael Berk, Orwa Dandash, Covadonga M. Díaz‐Caneja, Carl T. Edbom, Cecilie Johannessen, Anne‐Kathrin J. Fett, Sophia Frangou, Anthony C. James, Joost Janssen, Katherine H. Karlsgodt, Marinos Kyriakopoulos, Vera Lonning, Bjørn R. Rund, Runar E. Smelror, Alysha Sultan, Kirsten Wedervang‐Resell, Anne M. Myhre, Ole A. Andreassen, Ingrid Agartz, Ariana Vajdi, Lydia Krabbendam. Manuscript revision: Tiril P. Gurholt, Stener Nerland, Kjetil N. Jørgensen, Unn K. Haukvik, Claudia Barth, Carrie E. Bearden, Hannes Bohman, Michael Berk, Orwa Dandash, Covadonga M. Díaz‐Caneja, Anne‐Kathrin J. Fett, Sophia Frangou, Anthony C. James, Cecilie Johannessen, Michael Berk, Katherine H. Karlsgodt, Matthew J. Kempton, Marinos Kyriakopoulos, Neda Jahanshad, Bradley J. MacIntosh, Bjørn R. Rund, Runar E. Smelror, Christian K. Tamnes, Theo G. M. van Erp, Kirsten Wedervang‐Resell, Ole A. Andreassen, Paul M. Thompson, Ingrid Agartz, Lydia Krabbendam.

## Supporting information


**FIGURE S1** Violin plots of the participants age‐distribution split on early‐onset psychosis and healthy controls.
**FIGURE S2**: Violin plots of the participants ICV and combined subcortical structures split on early‐onset psychosis and healthy controls.
**FIGURE S3**: Effect of differences between early‐onset psychosis patients and typically developing adolescent controls, unadjusted for ICV.
**FIGURE S4**: Forest plots showing site‐wise differences between early‐onset psychosis patients and typically developing adolescent controls—combined structures.
**FIGURE S5**: Forest plots showing site‐wise differences between early‐onset psychosis patients and typically developing adolescent controls—left hemisphere.
**FIGURE S6**: Forest plots showing site‐wise differences between early‐onset psychosis patients and typically developing adolescent controls—right hemisphere.
**TABLE S1:** Cohort‐wise demographic and clinical information.
**TABLE S2**: Cohort‐wise inclusion and exclusion criteria.
**TABLE S3**: Scanner‐specific image acquisition and processing details.
**TABLE S4**: The effect of early‐onset psychosis patient status compared with typically developing adolescents.
**TABLE S5**: Follow‐up analyses for age‐by‐diagnosis interactions in early‐onset psychosis patients compared with controls.
**TABLE S6**: Follow‐up analyses for sex‐by‐diagnosis interactions in early‐onset psychosis patients compared with controls.
**TABLE S7**: The effect of early‐onset psychosis patient status compared with typically developing adolescents without ICV adjustment.
**TABLE S8**: Meta‐analytical result of early‐onset psychosis patients compared with typically developing adolescents after adjusting for sex, age, and ICV.
**TABLE S9**: The effect of patient subtype compared with typically developing adolescents—combined structures.
**TABLE S10**: The effect of patient subtype compared with typically developing adolescents—left structures.
**TABLE S11**: The effect of patient subtype compared with typically developing adolescents—right structures.
**TABLE S12**: Follow‐up analyses in patients for the effect of age at onset.
**TABLE S13**: Follow‐up analyses in patients for the effect of duration of illness.
**TABLE S14**: Antipsychotic users/non‐users compared to typically developing adolescents.
**TABLE S15**: Antipsychotic medication effects in patients only (reference antipsychotic medication non‐users).
**NOTE S1**: Image quality control.
**NOTE S2**: Meta analytical approach.

## Data Availability

The datasets from this study will not be made publicly available as we do not have approvals for sharing clinical data.

## Endnote

^1^
A meta‐analysis aggregates summary results (e.g., effect size estimates and confidence intervals) across studies, while a mega‐analysis aggregates individual participant data across cohorts. For an empirical comparison of the two techniques using structural MRI data, see Boedhoe et al. (2019).
