# Supplementary material for: Intracranial and subcortical volumes in adolescents with early‐onset psychosis: A multisite mega‐analysis from the ENIGMA consortium
Source: Hum Brain Mapp. 2020 Oct 5;43(1):373–84. doi: 10.1002/hbm.25212 (PMC8675418; doi:10.1002/hbm.25212)
Supplement: Supplementary file 1 — FIGURE S1 Violin plots of the participants age‐distribution split on early‐onset psychosis and healthy controls. FIGURE S2: Violin plots of the participants ICV and combined subcortical structures split on early‐onset psychosis and healthy controls. FIGURE S3: Effect of differences between early‐onset psychosis patients and typically developing adolescent controls, unadjusted for ICV. FIGURE S4: Forest plots showing site‐wise differences between early‐onset psychosis patients and typically developing adolescent controls—combined structures. FIGURE S5: Forest plots showing site‐wise differences between early‐onset psychosis patients and typically developing adolescent controls—left hemisphere. FIGURE S6: Forest plots showing site‐wise differences between early‐onset psychosis patients and typically developing adolescent controls—right hemisphere. TABLE S1: Cohort‐wise demographic and clinical information. TABLE S2: Cohort‐wise inclusion and exclusion criteria. TABLE S3: Scanner‐specific image acquisition and processing details. TABLE S4: The effect of early‐onset psychosis patient status compared with typically developing adolescents. TABLE S5: Follow‐up analyses for age‐by‐diagnosis interactions in early‐onset psychosis patients compared with controls. TABLE S6: Follow‐up analyses for sex‐by‐diagnosis interactions in early‐onset psychosis patients compared with controls. TABLE S7: The effect of early‐onset psychosis patient status compared with typically developing adolescents without ICV adjustment. TABLE S8: Meta‐analytical result of early‐onset psychosis patients compared with typically developing adolescents after adjusting for sex, age, and ICV. TABLE S9: The effect of patient subtype compared with typically developing adolescents—combined structures. TABLE S10: The effect of patient subtype compared with typically developing adolescents—left structures. TABLE S11: The effect of patient subtype compared with typically developing adolescents—right structures. TA [file HBM-43-373-s001.pdf]

Supplemental Information

Table of contents

SUPPLEMENTAL FIGURES 3

Figure S1: Violin plots of the participants age-distribution split on early-onset psychosis and healthy controls. 3

Figure S2: Violin plots of the participants ICV and combined subcortical structures split on early-onset psychosis and healthy controls. 4

Figure S3: Effect of differences between early-onset psychosis patients and typically developing adolescent controls, unadjusted for ICV. 5

Figure S4: Forest plots showing site-wise differences between early-onset psychosis patients and typically developing adolescent controls – combined structures. 6

Figure S5: Forest plots showing site-wise differences between early-onset psychosis patients and typically developing adolescent controls – left hemisphere. 7

Figure S6: Forest plots showing site-wise differences between early-onset psychosis patients and typically developing adolescent controls – right hemisphere. 8

SUPPLEMENTAL TABLES 9

Table S1. Cohort-wise demographic and clinical information. 9

Table S2. Cohort-wise inclusion and exclusion criteria. 11

Table S3. Scanner-specific image acquisition and processing details. 22

Table S4: The effect of early-onset psychosis patient status compared with typically developing adolescents. 24

Table S5: Follow-up analyses for age-by-diagnosis interactions in early-onset psychosis patients compared with controls. 25

Table S6: Follow-up analyses for sex-by-diagnosis interactions in early-onset psychosis patients compared with controls. 26

Table S7: The effect of early-onset psychosis patient status compared with typically developing adolescents without ICV adjustment. 27

Table S8: Meta-analytical result of early-onset psychosis patients compared with typically developing adolescents after adjusting for sex, age and ICV. 28

Table S9: The effect of patient subtype compared with typically developing adolescents - combined structures. 29

Table S10: The effect of patient subtype compared with typically developing adolescents - left structures. 30

Table S11: The effect of patient subtype compared with typically developing adolescents - right structures. 31

Table S12: Follow-up analyses in patients for the effect of age at onset. 32

Table S13: Follow-up analyses in patients for the effect of duration of illness. 33

Table S14: Antipsychotic users/non-users compared to typically developing adolescents. 34

Table S15: Antipsychotic medication effects in patients only (reference antipsychotic medication non-users). 35

SUPPLEMENTAL METHOD 36

Note S1: Image quality control 36

Note S2: Meta analytical approach 36

REFERENCES 37

Supplemental Figures

Figure S1: Violin plots of the participants age-distribution split on early-onset psychosis and healthy controls.

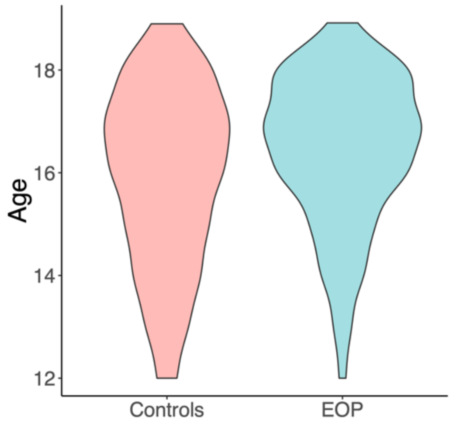

Abbreviations: EOP: early-onset psychosis.

Figure S2: Violin plots of the participants ICV and combined subcortical structures split on early-onset psychosis and healthy controls.

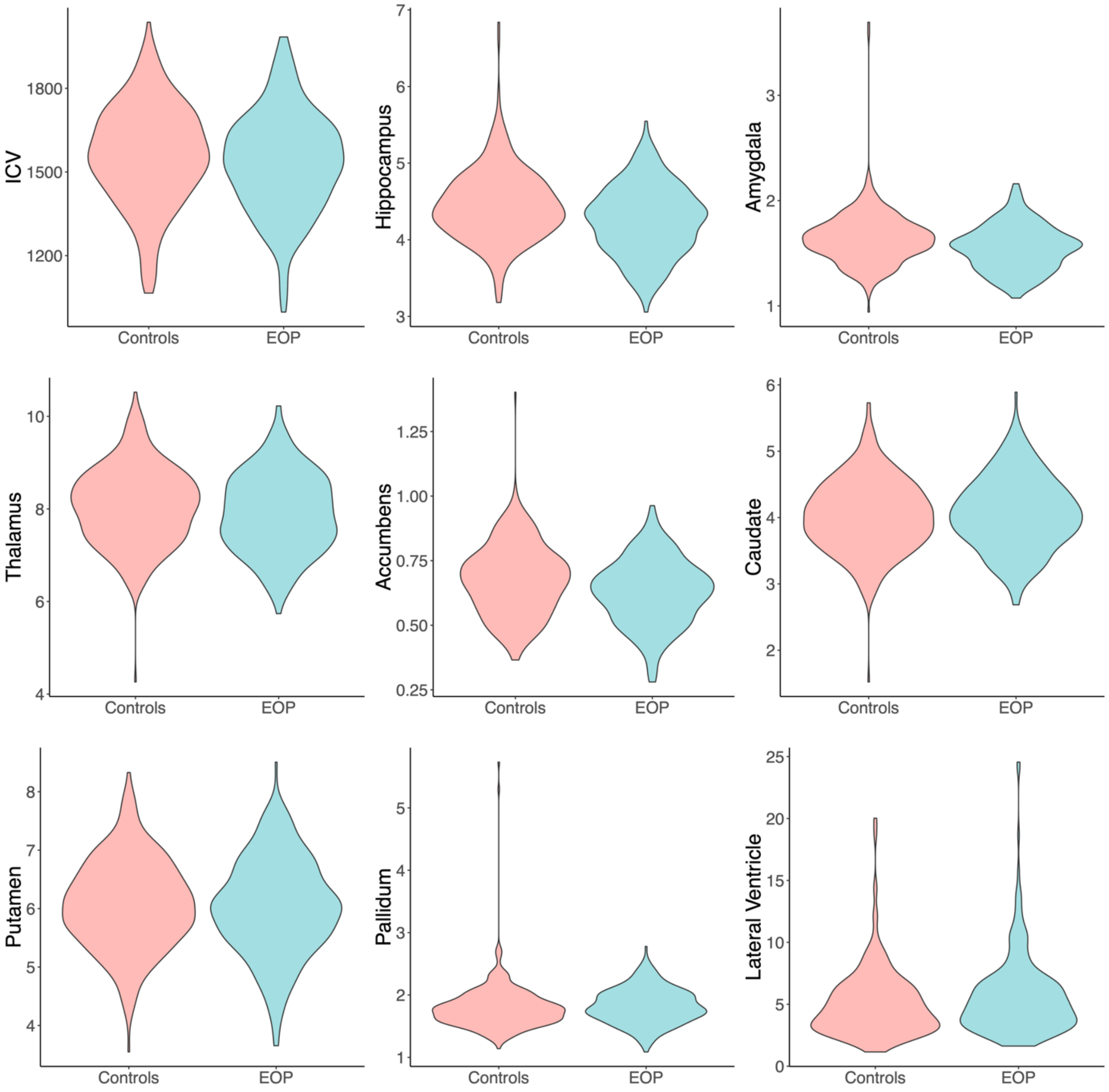

Notes: Distributions of ICV and subcortical volumes are presented in ml. *Abbreviations:* EOP: early-onset psychosis, ICV: intracranial volume.

Figure S3: Effect of differences between early-onset psychosis patients and typically developing adolescent controls, unadjusted for ICV.

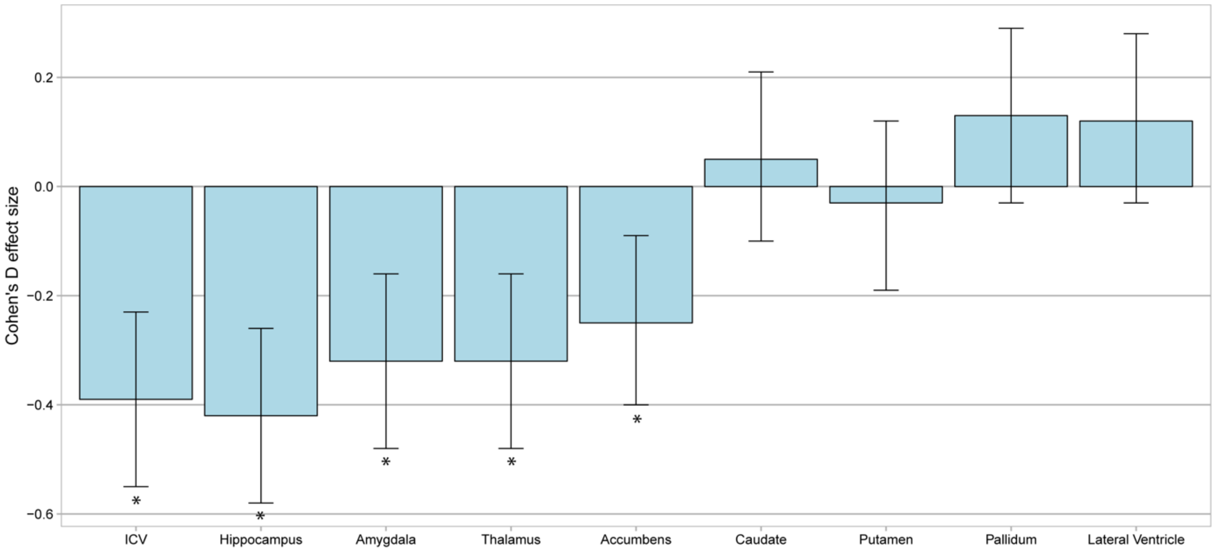

Notes: Linear mixed-effects models unadjusted for ICV applied for diagnostic differences between early-onset psychosis patients and controls (reference). We adjusted for age and sex as fixed-effects variables, and scanner as a random-effects variable. Error bars show mean± standard error of effect size. Significant differences indicated by *.

Figure S4: Forest plots showing site-wise differences between early-onset psychosis patients and typically developing adolescent controls – combined structures.

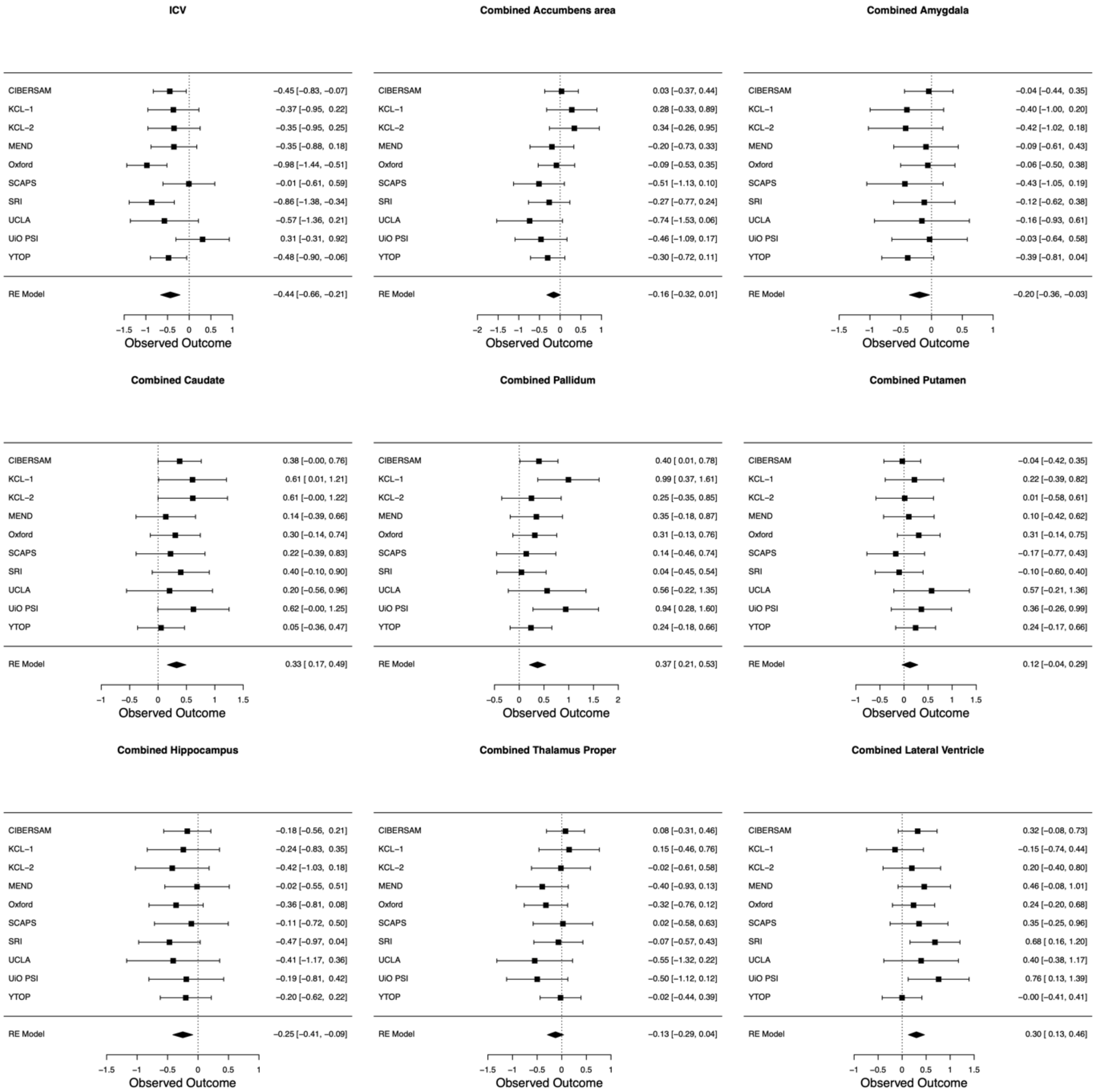

Notes: Linear regression models applied for diagnostic differences between early-onset psychosis patients and controls (reference) at each site, adjusting for age, sex and ICV (for subcortical structures) as covariate. For cohort with multiple scanners, scanner was a covariate. For each cohort, the Cohen's d effect size and 95% confidence interval are shown. The diamond indicates the pooled effect size across cohorts.

Figure S5: Forest plots showing site-wise differences between early-onset psychosis patients and typically developing adolescent controls – left hemisphere.

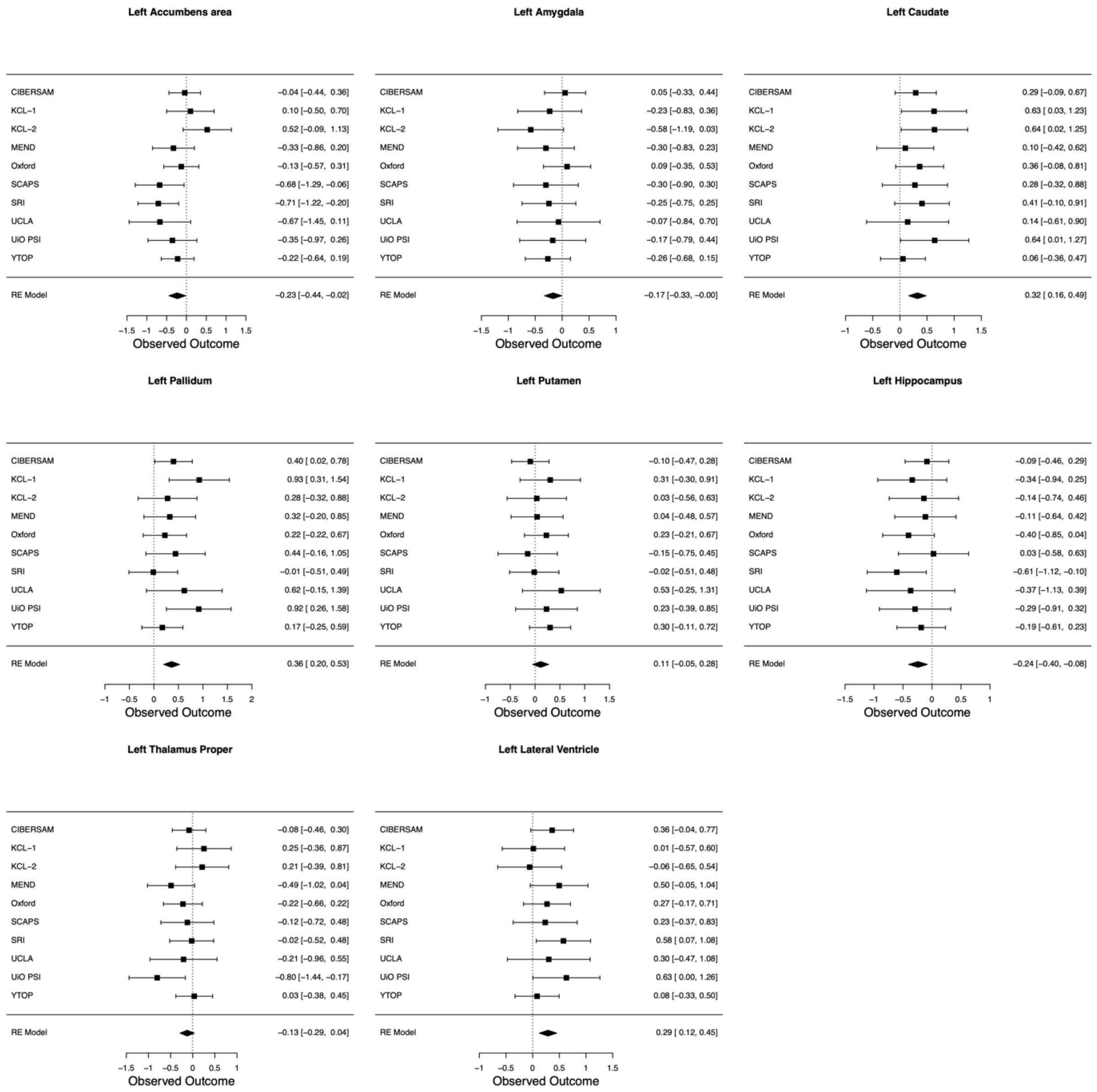

Notes: Linear regression models applied for diagnostic differences between early-onset psychosis patients and controls (reference) at each site. We adjusted for age, sex and ICV (for subcortical structures) as covariate. For sites with multiple scanners, scanner was a covariate. For each cohort, the Cohen's d effect size and 95% confidence interval are shown. The diamond indicates the pooled effect size across cohorts.

Figure S6: Forest plots showing site-wise differences between early-onset psychosis patients and typically developing adolescent controls – right hemisphere.

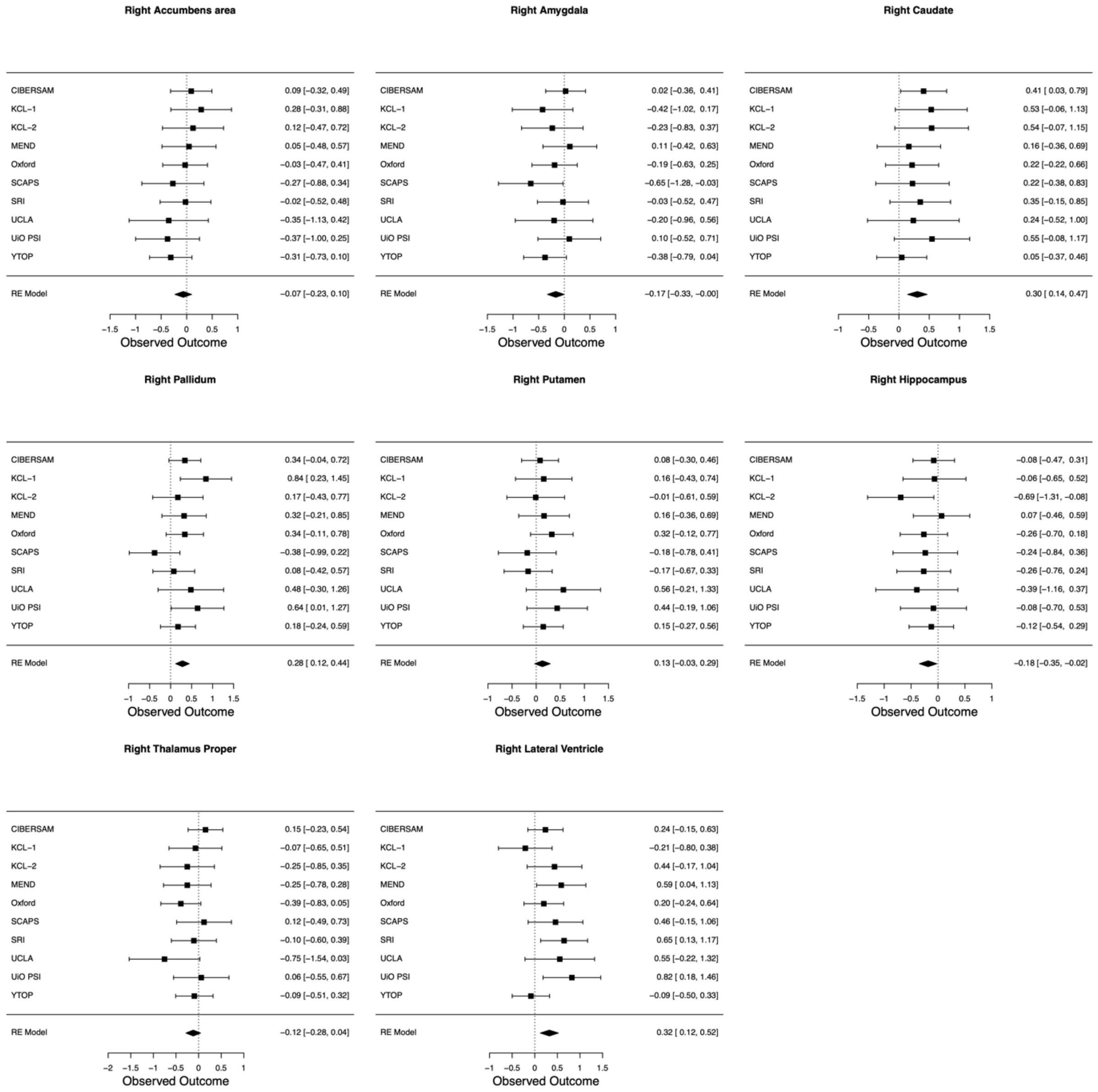

Notes: Linear regression models applied for diagnostic differences between early-onset psychosis patients and controls (reference) at each site. We adjusted for age, sex and ICV (for subcortical structures) as covariate. For sites with multiple scanners, scanner was a covariate. For each cohort, the Cohen's d effect size and 95% confidence interval are shown. The diamond indicates the pooled effect size across cohorts.

Supplemental Tables

Table S1. Cohort-wise demographic and clinical information.

	**Number of participants**					**Female N (%)**		**Age**		**Patient specific measures**					
**Cohort**	**All**	**EOP**	**HC**	**EOS/AFP/OTP**	**AP user/non-user**	**EOP**	**HC**	**EOP**	**HC**	**AAO**	**DOI**	**PANSS Pos.**	**PANSS Neg.**	**SANS**	**SAPS**
**CIBERSAM**	111	49	62	44/5/0	49/0	13 (26.5)	21 (33.9)	16.2±1.4	14.8±1.8	15.7±1.4	.4±.4	24.2±8.8	24.2±8.9		
**KCL-2**	45	20	25	6/2/12	17/3	0 (0)	0 (0)	16.9±1.2	16.4±1.6	16.3±1.4	1.1±.8				
**Deakin**	6	4	2	0/4/0	1/3	1 (25)	1 (50)	17.8±0.5	16±0						
**KCL-1**	47	29	18	29/0/0	0/0	10 (34.5)	7 (38.9)	16.3±1.7	16.1±1.5	14.8±2.4	1.6±1.4	10±3.1	12.6±3.3		
**SCAPS**	45	23	22	2/14/7	16/6	10 (43.5)	17 (77.3)	16.4±1.3	16.9±1.3	15.3±2.2	1.1±1.6				
**MEND**	58	16	42	11/2/3	16/0	7 (43.8)	21 (50)	17.9±.7	16.4±2.1	16.3±1.2	1.7±0.9				
**Oxford**	82	44	38	44/0/0	44/0	18 (40.9)	20 (52.6)	16.3±1.2	16.2±1.4	14.6±1.5	1.8±1.3	22.5±2.9	16.1±3.2		

**SRI**	64	10	54	0/10/0	8/2	6 (60)	29 (53.7)	17.4±1	16.4±1.3	15±1.7	2.4±1.8				
**UCLA**	29	15	14	15/0/0	11/3	5 (33.3)	5 (35.7)	15.7±1.8	14.9±2.1	14.1±1.8	1.5±1.5			30.6±10.5	19.7±23.3
**YTOP**	92	35	57	20/2/13	19/16	25 (71.4)	31 (54.4)	16.5±1.2	15.9±1.6	14.7±1.8	1.8±1.6	16.8±4.8	18.9±7.3		
**UiO PSI**	43	18	25	12/0/6	12/6	8 (44.4)	11 (44)	15.7±1.8	15.5±1.5	13.8±2.3	2.5±1.9	13.4±4.5	12.7±3.8		

Full name of cohorts: CIBERSAM, Madrid, KCL-2: King's College London (Cohort 2). Deakin: Deakin University, KCL-1: King's College London (cohort 1), SCAPS: Stockholm Child and Adolescent Psychosis Study, MEND: Multimodal Assessment of Neurodevelopmental Disorders, Oxford: Oxford University, SRI: Sunnybrook Research Institute, UCLA: University of California, Los Angeles, YTOP: Thematically Organized Psychosis Study for Youth, UiO PSI: University of Oslo, Department of Psychology. *Abbreviations:* AAO: Age at onset, AFP: affective psychosis, AP: antipsychotics, DOI: Duration of Illness, EOP: early-onset psychosis, EOS: early-onset schizophrenia, HC: healthy controls, OTP: other psychosis, PANSS: Positive and Negative Syndrome Scale, SANS: Scale for the Assessment of Negative Symptoms, SAPS: Scale for the Assessment of Positive Symptoms.

Table S2. Cohort-wise inclusion and exclusion criteria.

Cohort	Diagnosis	Instrument for clinical assessment	Recruitment information	Inclusion criteria	Exclusion criteria
**CIBERSAM**	Patient	K-SADS	Local clinical services referred to a specialized early-onset psychosis program PIENSA or hospitalized in an adolescent inpatient Unit (Hospital General Universitario Gregorio Marañón)	Inclusion criteria for patients consisted of (1) age between 7-18 years, (2) onset of the first psychotic positive symptom within a psychotic episode before the age of 18, (3) diagnosis of a psychotic disorder per DSM-IV-TR criteria, (4) written informed consent	Exclusion criteria for patients consisted of: (1) intellectual disability per DSM-IV-TR criteria (IQ<70 and impaired functioning), (2) pervasive developmental disorder, (3) personal history of head trauma with loss of consciousness, and (4) pregnancy.
	Control	K-SADS	Local area via advertisements	Inclusion criteria for controls consisted of (1) age between 7-18 years, (2) written informed consent	Exclusion criteria for controls consisted of: (1) personal history of psychotic illness, (2) current diagnosis of any Axis-I DSM-IV-TR disorder, (3) intellectual disability per DSM-IV-TR criteria (IQ<70 and impaired functioning), (4) past head trauma with loss of consciousness, and (5) pregnancy.

**KCL-2**	Patient	ICD-10 diagnosed by treating clinician	Patients were recruited via consultant psychiatrists and via the Mental Health Research Network (MHRN) in South London and Maudsley-, North East London- and South Essex Partnership University NHS Foundation Trusts.	Inclusion criteria for patients consisted of (1) age between 13-19 years, (2) experienced a psychotic episode according to ICD-10 criteria, as diagnosed by their clinician, (3) good command of the English language and (4) being able and willing to give written informed consent.	
	Control	No dx confirmed in telephone screening	Control participants were recruited from local schools, the Institute of Psychiatry volunteer database 'Mindsearch', via colleagues and previous participants.	Inclusion criteria for the control group were (1) age between 13-19 years, (2) good command of the English language and (3) able and willing to give written informed consent, and (4) no personal or family history of a psychotic illness.	
**KCL-1**	Patient	Structured Clinical Interview for DSM-IV (SCID) Axis I Disorders	Local clinical services	DSM IV Schizophrenia, 12 - 19 years old, onset of schizophrenia before age 18, no comorbid Axis I diagnosis, no mental retardation	History of head injury, current substance misuse, any medical condition, history of hereditary disease of the nervous system

	Control	Structured Clinical Interview for DSM-IV (SCID) Axis I Disorders	Local area via advertisements	Age 12 - 19, no personal history of psychiatric disorder, no family history of psychosis in first degree relatives	History of head injury, current substance misuse, any medical condition, history of hereditary disease of the nervous system
**SCAPS**	Patient		Specialist care unit of psychosis and bipolar disorder in the department of Child and Adolescent Psychiatry in Stockholm, Sweden.	Diagnoses according to DSM-IV. Psychotic disorder diagnoses included schizophrenia, schizoaffective disorder, psychotic depression and unspecified psychosis. Bipolar disorder included bipolar 1 and 2 disorders.	Diagnosis of substance-induced psychotic disorder. IQ<70. Previous moderate to severe head injury. Organic brain disease.
	Control		Random draw from the Swedish National Registry and invited by letter to participate	Age 12-18 years. Swedish language abilities to complete interview and neurocognitive tests	History of mental health issues warranting contact with specialist mental health services. Previous or current use of psychotrophic medication. No first-degree relative with a history of psychotic disorder. IQ < 70. Previous moderate to severe head injury. Organic brain disease.

**MEND**	Patient	SCID for DSM-IV	Recruited through inpatient units at Zucker Hillside Hospital (Glen Oaks, NY) and physician referral	Age 12-25 years (for full study), within 2 years of onset, currently treated on atypical antipsychotics, sufficiently fluent in English to avoid invalidating neuropsychological measures, able to complete MRI	Psychosis due to substance use or general medical condition, neurological disorders, substance use disorder in last 6 months, MRI contraindications (head injury, claustrophobia, metal, etc)
	Control	SCID for DSM-IV	Recruited through community advertisement	Age 12-25 (for full study), sufficiently fluent in English to avoid invalidating neuropsychological measures, able to complete MRI	First degree relative with a diagnosis of a psychotic or mood disorder, neurological disorders, substance use disorder in last 6 months, medications affecting cognitive function, MRI contraindications (head injury, claustrophobia, metal, etc)
**Deakin**	Patient	Meet DSM-IV criteria for mania as part of bipolar I disorder or schizoaffective disorder. (SCID-P). Montgomery Asberg Depression Rating Scale (MADRS). Bipolar Depression	Within ORYGEN, the Youth Access Team (YAT) functions as the gate-keeper. Patients were referred to the YAT by the Triage team.	Not have had a previous treated manic episode. Have the capacity to provide informed consent to the study and comply with study procedures. Be utilizing effective contraception if female, sexually	Patients with a known or suspected clinically relevant systemic medical disorder. Individuals who are pregnant or lactating. Patients who have had a prior sensitivity or allergy to

		Rating Scale (BDRS). Young Mania Rating Scale (YMRS). Clinical Global Impression (CGI) improvement and severity scales. CGI modified bipolar disorder (CGI BP). Global Assessment of Functioning Scale (GAF). Social and Occupational Functioning Assessment Scale (SOFAS). Premorbid Adjustment Scale (PAS).	Within the Geelong and the Southern Health sites, individuals were recruited from the early psychosis service (EPS) by a dedicated Case Manager researcher.	active and of childbearing age. Patients will need to have been on quetiapine and lithium as standard therapy for at least 1 month prior to randomization. Male or female patients age 15 to 25 years.	quetiapine, lithium or their components. Inability to comply with either the requirements of informed consent or the treatment protocol. Non-fluency in English. History of epilepsy. Clinically relevant biochemical or haematological abnormalities at baseline. Patients at immediate risk of self-harm or risk to others. Organic mental disease, including mental retardation (Full scale IQ<70). Use of any of the following cytochrome P450 3A4 inhibitors in the 14 days preceding enrolment including but not limited to: ketoconazole, itraconazole, fluconazole, erythromycin, clarithromycin, troleandomycin, indinavir, nelfinavir, ritonavir, fluvoxamine and saquinavir. Use of any of the

					following cytochrome P450 inducers in the 14 days preceding enrollment including but not limited to: phenytoin, carbamazepine, barbiturates, rifampin, St. John's Wort, and glucocorticoids. An absolute neutrophil count (ANC) of £1.5 x 10^9 per liter. Uncontrolled Diabetes Mellitus (DM).
	Control	NA	Control subjects were recruited via advertising and friends of patients frequenting psychiatric services outlined above. Recruitment was additionally made by the Melbourne Neuropsychiatry Centre (MNC), Parkville, Melbourne.	No history of mental illness and matched to patient participants on demographic characteristics. Have the capacity to provide informed consent to the study and comply with study procedures. Be utilizing effective contraception if female, sexually active and of childbearing age. Male or female participants age 15 to 25 years.	Similar to Patients

**Oxford**	Patient	KSADS-PL	Local adolescent psychiatric units	DSM IV Schizophrenia	Exclusion criteria included moderate mental impairment (IQ 560), a history of substance abuse or pervasive developmental disorder, significant head injury, neurological disorder or major medical disorder
	Control	KSADS-PL	Local GP practices	Healthy adolescents	Exclusion criteria included any medical emotional behavioral disorders, moderate mental impairment (IQ 560), a history of substance abuse or pervasive developmental disorder, significant head injury, neurological disorder or major medical disorder
**SRI**	Patient	KSADS-PL	Centre for Youth Bipolar Disorder	English-speaking, of any race/ethnicity, and 13-20 years of age meeting diagnostic criteria for BD (type I, II, or NOS),	1) unable to provide informed consent, 2) existing cardiac condition (e.g. conduction abnormality or congenital heart disease), auto-immune illness, or

					inflammatory illness, 3) currently taking anti-inflammatory, anti-lipidemic, anti-hypertensive agents 4) contraindications to magnetic resonance imaging (e.g. cardiac pacemaker or other implanted device) 5) neurological or cognitive impairment, 6) infectious illness within the past 14 days, 7) substance dependence in the past 3 months
	Control	KSADS-PL	Community via advertisement	English-speaking, of any race/ethnicity, and 13-20 years of age with no major psychiatric disorders and no family history of BD.	1) unable to provide informed consent, 2) existing cardiac condition (e.g. conduction abnormality or congenital heart disease), auto-immune illness, or inflammatory illness, 3) currently taking anti-inflammatory, anti-lipidemic, anti-hypertensive agents 4) contraindications to magnetic resonance imaging (e.g. cardiac pacemaker or other

					implanted device) 5) neurological or cognitive impairment, 6) infectious illness within the past 14 days, 7) substance dependence in the past 3 months.
**UCLA**	Patient	Structured Clinical Interview for DSM-IV (SCID) Axis I Disorders	Recruited from clinicians at in- and outpatient child- and adolescent mental health clinics in the Greater Los Angeles area/website/local advertisements	Diagnosis of schizophrenia spectrum disorder. Have the capacity to provide informed consent to the study. No MRI contra-indications. Male or female participants age 12 to 18 years.	Diagnosis of substance-induced psychotic disorder. IQ<70. Previous moderate to severe head injury. Significant comorbid medical or neurologic condition and/or history of head trauma with loss of consciousness.
	Control	Structured Clinical Interview for DSM-IV (SCID) Axis I Disorders	Online postings and brochures in the Los Angeles area	No history of major mental disorder and matched to patient participants on demographic characteristics. Have the capacity to provide informed consent to the study. No MRI contra-indications. Male or female participants age 12 to 18 years.	History of mental health issues warranting contact with specialist mental health services. No first-degree relative with a history of psychotic disorder. IQ < 70. Significant comorbid medical or neurologic condition and/or history of head trauma with loss of consciousness.

**YTOP** [Wedervang-Resell et al., 2019]	Patient	KSADS-PL version 2009	Recruited from clinicians at in- and outpatient child- and adolescent mental health clinics in the Greater Oslo area	Age 12-18 years. DSM-IV verified diagnosis of psychotic disorder. Norwegian language abilities to complete diagnostic interview and neurocognitive tests	Diagnosis of substance-induced psychotic disorder. IQ<70. Previous moderate to severe head injury. Organic brain disease.
	Control	KSADS-PL version 2009	Random draw from the Norwegian National Registry and invited by letter to participate.	Age 12-18 years. Norwegian language abilities to complete diagnostic interview and neurocognitive tests	History of mental health issues warranting contact with specialist mental health services. Previous or current use of psychotropic medication. First-degree relative with a history of psychotic disorder. IQ < 70. Previous moderate to severe head injury. Organic brain disease.
**UiO PSI**	Patient	SCID-I, mod A-D; PANSS; GAF Split version	Recruited from clinicians at in- and outpatient child- and adolescent mental health clinics in Southern Norway	Age 12-18. Broad schizophrenia spectrum disorder.	Psychosis NOS. History of head injury. IQ<70
	Control	Screened with MINI, screening mod	Recruited from schools in the patient catchment area, plus some from the database		

			of the Norwegian Central Bureau of Statistics		

Notes: Reference given at the end of document.

Table S3. Scanner-specific image acquisition and processing details.

	Scanner	Sequence	Field Strength	Acquisition Direction	Number of Slices	Slice Spacing	Voxel Size (mm^3)	TI	TE	TR	Flip Angle
**CIBERSAM**	**1**	3D T1-weighted	1.5T Philips	Sagittal	175		1x0.94x0.94		9.2	25	
	**2**	3D T1-weighted	3.0T General Electric	Sagittal	196		1x1x1.2	400	0 (MinFull)	6.5	11
**KCL-2**	**1**		3T GE Signa	Coronal	196		1.1x1.1x1.1		2.8	7	20
**KCL-1**	**1**	3D T1-weighted	GE 1.5 Tesla	Coronal	124	1.5	0.86x0.86x1.5	450	3.0	14	20
	**2**	3D T1-weighted	GE Signa HDx 1.5 Tesla	Axial	146	1.1	1.1x1.1.x1.1	300	4.8	11.2	18
**SCAPS**	**1**		3T GE Discovery MR 750	Sagittal			1x1x1.2		3.06	7.98	12
**MEND**	**1**	3D T1-weighted	3T Siemens Verio	Axial	155		1x1x1	1100	3.3	2530	7
**Deakin**	**1**	3D T1- weighted	3T Siemens TrioTim	Sagittal	192		.9x.9x.9	900ms	2.24ms	2000ms	9

	**2**	3D T1- weighted	3T Siemens TrioTim	Sagittal	192		.5x.5x.9	900ms	2.24ms	2000ms	9
**Oxford**	**1**		1.5T siemens	Coronal	208		1x1x1		5.6	12	19
**SRI**	**1**	3D T1-weighted fast spoiled gradient echo imaging	3T Philips Achieva	Axial	142	1mm	1x1x1	650ms	3.2ms	8.1ms	8
**UCLA** [Chung et al., 2018; Eckfeld et al., 2017]	**1**†	3D T1-weighted	3T Siemens Tim Trio	Sagittal	192	1.2 mm	1x1x1	900ms	2.91ms	2300ms	9
**YTOP** [Gurholt et al., 2018; Tesli et al., 2020]	**1**	3D T1-weighted fast spoiled gradient echo (FSPGR) sequence	3T GE Signa HDxt	Sagittal	166	1.2	1x1x1.2	450	MinFull	7.8	12
	**2**	BRAVO sequence	3T GE Discovery MR 750	Sagittal	188	1	1x1x1	450	3.18	8.16	12
**UiO PSI** [Juuhl-Langseth et al., 2012]	**1**	3D Spoiled Gradient Recalled (3D-SPGR)	1.5T Siemens Sonata Vision	Coronal	160	1	1x1x1	1000	3.93	2730	7

Notes: Reference given at the end of document.

† At the UCLA site, 7 subjects were scanned at the Brain Mapping Center's 3.0 T Siemens Tim Trio, using the identical protocol, head coil and software. Site was adjusted for in the analyses.

Table S4: The effect of early-onset psychosis patient status compared with typically developing adolescents.

	**Combined structures**			**Left hemisphere**			**Right hemisphere**		
**Structure**	**Cohen's d**	**t-value**	**p-value**	**Cohen's d**	**t-value**	**p-value**	**Cohen's d**	**t-value**	**p-value**
ICV	−0.39	−4.75	**3e−06**						
Hippocampus	−0.25	−2.99	**0.0029**	−0.24	−2.85	**0.0045**	−0.19	−2.27	0.0238
Amygdala	−0.20	−2.34	0.0199	−0.18	−2.21	0.0272	−0.15	−1.78	0.0760
Thalamus	−0.13	−1.54	0.1240	−0.13	−1.58	0.1154	−0.12	−1.45	0.1462
Accumbens	−0.11	−1.34	0.1820	−0.14	−1.62	0.1065	−0.06	−0.71	0.4796
Caudate	0.25	3.07	**0.0022**	0.27	3.29	**0.0010**	0.23	2.72	0.0067
Putamen	0.16	1.89	0.0594	0.15	1.78	0.0764	0.15	1.79	0.0747
Pallidum	0.24	2.89	**0.0040**	0.28	3.37	**8e−04**	0.16	1.88	0.0599
Lateral Ventricle	0.22	2.60	0.0095	0.24	2.88	**0.0042**	0.20	2.42	0.0157

Notes: Results of linear-mixed effect model for diagnostic (patient-control) differences (fixed factors: diagnosis, age, sex and ICV; random factor: scanner). *Abbreviations:* ICV: intracranial volume.

Table S5: Follow-up analyses for age-by-diagnosis interactions in early-onset psychosis patients compared with controls.

	Combined structures			Left hemisphere			Right hemisphere		
**Structure**	**Cohen's d**	**t-value**	**p-value**	**Cohen's d**	**t-value**	**p-value**	**Cohen's d**	**t-value**	**p-value**
ICV	0.02	0.30	0.7662						
Hippocampus	0.02	0.29	0.7691	0.05	0.57	0.5687	0.01	0.15	0.8805
Amygdala	0.01	0.16	0.8716	−0.02	−0.21	0.8339	0.03	0.34	0.7351
Thalamus	−0.02	−0.29	0.7742	0	−0.02	0.9858	−0.07	−0.86	0.3897
Accumbens	0.05	0.64	0.5242	0.07	0.89	0.3719	0.04	0.46	0.6424
Caudate	0.05	0.60	0.5510	0.05	0.63	0.5311	0.05	0.65	0.5153
Putamen	0.07	0.81	0.4210	0.06	0.73	0.4642	0.07	0.86	0.3900
Pallidum	0.10	1.26	0.2084	0.09	1.05	0.2931	0.11	1.31	0.1922
Lateral Ventricle	0.08	0.93	0.3545	0.06	0.71	0.4760	0.12	1.45	0.1479

Notes: Results of linear-mixed effect model for age-by-diagnosis interaction effects (fixed factors: diagnosis, age-by-diagnosis, age, sex and ICV; random factor: scanner). *Abbreviations:* ICV: intracranial volume.

Table S6: Follow-up analyses for sex-by-diagnosis interactions in early-onset psychosis patients compared with controls.

	**Combined structures**			**Left hemisphere**			**Right hemisphere**		
**Structure**	**Cohen's d**	**t-value**	**p-value**	**Cohen's d**	**t-value**	**p-value**	**Cohen's d**	**t-value**	**p-value**
ICV	−0.08	−1.01	0.3118						
Hippocampus	0.01	0.10	0.9172	0	0.03	0.9731	0.02	0.22	0.8274
Amygdala	0.01	0.11	0.9115	0.06	0.76	0.4480	−0.04	−0.46	0.6421
Thalamus	0.02	0.24	0.8106	0	0.04	0.9675	0.03	0.36	0.7172
Accumbens	−0.10	−1.20	0.2303	−0.06	−0.69	0.4927	−0.11	−1.30	0.1947
Caudate	−0.16	−1.96	0.0508	−0.16	−1.95	0.0514	−0.16	−1.86	0.0639
Putamen	−0.14	−1.64	0.1014	−0.13	−1.55	0.1226	−0.10	−1.16	0.2446
Pallidum	−0.05	−0.55	0.5813	−0.04	−0.53	0.5947	−0.04	−0.48	0.6322
Lateral Ventricle	−0.05	−0.56	0.5779	−0.01	−0.15	0.8843	−0.08	−0.94	0.3454

Notes: Results of linear-mixed effect model for sex-by-diagnosis interaction effects (fixed factors: diagnosis, sex-by-diagnosis, age, sex and ICV; random factor: scanner). *Abbreviations:* ICV: intracranial volume.

Table S7: The effect of early-onset psychosis patient status compared with typically developing adolescents without ICV adjustment.

	**Combined structures**			**Left hemisphere**			**Right hemisphere**		
**Structure**	**Cohen's d**	**t-value**	**p-value**	**Cohen's d**	**t-value**	**p-value**	**Cohen's d**	**t-value**	**p-value**
ICV	−0.39	−4.75	**3e−06**						
Hippocampus	−0.42	−5.04	**6e−07**	−0.39	−4.71	**3e−06**	−0.36	−4.32	**2e−05**
Amygdala	−0.32	−3.82	**2e−04**	−0.30	−3.60	**4e−04**	−0.27	−3.24	**0.0013**
Thalamus	−0.32	−3.88	**1e−04**	−0.33	−3.94	**9e−05**	−0.31	−3.73	**2e−04**
Accumbens	−0.25	−2.93	**0.0035**	−0.24	−2.90	**0.0039**	−0.19	−2.21	0.0277
Caudate	0.05	0.65	0.5164	0.07	0.91	0.3642	0.04	0.47	0.6388
Putamen	−0.03	−0.42	0.6763	−0.03	−0.42	0.6777	−0.03	−0.35	0.7247
Pallidum	0.13	1.59	0.1126	0.19	2.26	0.0241	0.06	0.73	0.4677
Lateral Ventricle	0.12	1.49	0.1379	0.14	1.71	0.0877	0.13	1.51	0.1323

Notes: Results of linear-mixed effect model for diagnostic (patient-control) differences (fixed factors: diagnosis, age and sex; random factor: scanner). *Abbreviations:* ICV: intracranial volume.

Table S8: Meta-analytical result of early-onset psychosis patients compared with typically developing adolescents after adjusting for sex, age and ICV.

	**Combined structures**			**Left hemisphere**			**Right hemisphere**		
**Structure**	**Cohen's d**	**z-value**	**p-value**	**Cohen's d**	**z-value**	**p-value**	**Cohen's d**	**z-value**	**p-value**
ICV	−0.44	−3.83	**0.0001**						
Hippocampus	−0.25	−3.01	**0.0027**	−0.24	−2.91	**0.0036**	−0.18	−2.22	0.0261
Amygdala	−0.20	−2.34	0.0191	−0.17	−2.02	0.0435	−0.17	−2.01	0.0447
Thalamus	−0.13	−1.52	0.1278	−0.13	−1.52	0.1295	−0.12	−1.43	0.1538
Accumbens	−0.16	−1.86	0.0624	−0.23	−2.15	0.0319	−0.07	−0.79	0.4300
Caudate	0.33	3.95	**8e−05**	0.32	3.91	**9e−05**	0.3	3.68	**0.0002**
Putamen	0.12	1.49	0.1368	0.11	1.37	0.1715	0.13	1.57	0.1153
Pallidum	0.37	4.43	**1e−05**	0.36	4.34	**1e−05**	0.28	3.39	**0.0007**
Lateral Ventricle	0.30	3.51	**0.0005**	0.29	3.44	**0.0006**	0.32	3.16	**0.0016**

Notes: Meta-analytical results for diagnostic (patient-control) differences (covariates: diagnosis, age, sex and ICV (and scanner if multiple scanners at site)).

Abbreviations: ICV: intracranial volume.

Table S9: The effect of patient subtype compared with typically developing adolescents - combined structures.

	**Early-onset schizophrenia**			**Affective psychosis**			**Other psychosis**		
**Structure**	**Cohen's d**	**t-value**	**p-value**	**Cohen's d**	**t-value**	**p-value**	**Cohen's d**	**t-value**	**p-value**
ICV	−0.34	−3.91	**1e−04**	−0.42	−3.03	**0.0025**	−0.26	−1.95	0.0515
Hippocampus	−0.24	−2.75	**0.0061**	−0.23	−1.67	0.0947	−0.13	−0.92	0.3582
Amygdala	−0.19	−2.20	0.0285	−0.31	−2.23	0.0264	0.03	0.24	0.8108
Thalamus	−0.19	−2.16	0.0313	−0.02	−0.13	0.8969	0.05	0.38	0.7020
Accumbens	−0.06	−0.65	0.5190	−0.29	−2.04	0.0414	−0.05	−0.38	0.7046
Caudate	0.19	2.19	0.0291	0.24	1.77	0.0773	0.31	2.27	0.0237
Putamen	0.15	1.75	0.0815	−0.02	−0.13	0.8939	0.23	1.68	0.0929
Pallidum	0.29	3.34	**9e−04**	0.05	0.35	0.7239	0.09	0.64	0.5206
Lateral Ventricle	0.14	1.57	0.1159	0.32	2.27	0.0238	0.27	1.99	0.0475

Notes: Results of linear-mixed effect model for patient subtype (EOS/AFP/OTP) with controls (reference) (fixed factors: diagnosis, age, sex and ICV; random factor: scanner). *Abbreviations:* ICV: intracranial volume.

Table S10: The effect of patient subtype compared with typically developing adolescents - left structures.

	**Early-onset schizophrenia**			**Affective psychosis**			**Other psychosis**		
**Structure**	**Cohen's d**	**t-value**	**p-value**	**Cohen's d**	**t-value**	**p-value**	**Cohen's d**	**t-value**	**p-value**
Hippocampus	−0.23	−2.66	**0.0080**	−0.22	−1.60	0.1102	−0.11	−0.79	0.4288
Amygdala	−0.19	−2.15	0.0320	−0.29	−2.11	0.0357	0.05	0.38	0.7058
Thalamus	−0.17	−1.93	0.0542	−0.08	−0.60	0.5501	0.03	0.25	0.8026
Accumbens	−0.09	−1.02	0.3101	−0.39	−2.80	**0.0052**	0.04	0.32	0.7492
Caudate	0.18	2.08	0.0376	0.32	2.36	0.0188	0.35	2.59	0.0098
Putamen	0.13	1.46	0.1437	−0.01	−0.10	0.9234	0.26	1.93	0.0543
Pallidum	0.28	3.23	**0.0013**	0.22	1.62	0.1060	0.13	0.99	0.3221
Lateral Ventricle	0.16	1.84	0.0665	0.37	2.64	0.0085	0.25	1.82	0.0687

Notes: Results of linear-mixed effect model for patient subtype (EOS/AFP/OTP) with controls (reference) (fixed factors: diagnosis, age, sex and ICV; random factor: scanner). *Abbreviations:* ICV: intracranial volume.

Table S11: The effect of patient subtype compared with typically developing adolescents - right structures.

	**Early-onset schizophrenia**			**Affective psychosis**			**Other psychosis**		
**Structure**	**Cohen's d**	**t-value**	**p-value**	**Cohen's d**	**t-value**	**p-value**	**Cohen's d**	**t-value**	**p-value**
Hippocampus	−0.17	−1.99	0.0468	−0.19	−1.36	0.1757	−0.11	−0.79	0.4287
Amygdala	−0.13	−1.52	0.1297	−0.27	−1.92	0.0550	0.01	0.08	0.9336
Thalamus	−0.20	−2.26	0.0244	0.05	0.36	0.7211	0.05	0.40	0.6903
Accumbens	0	−0.05	0.9581	−0.11	−0.82	0.4148	−0.14	−1.05	0.2964
Caudate	0.18	2.12	0.0348	0.14	1.0	0.3168	0.29	2.17	0.0303
Putamen	0.16	1.84	0.0663	−0.02	−0.12	0.9014	0.16	1.18	0.2373
Pallidum	0.24	2.74	**0.0062**	−0.11	−0.79	0.4323	0.02	0.17	0.8631
Lateral Ventricle	0.14	1.57	0.1180	0.20	1.46	0.1449	0.32	2.37	0.0179

Notes: Results of linear-mixed effect model for patient subtype (EOS/AFP/OTP) with controls (reference) (fixed factors: diagnosis, age, sex and ICV; random factor: scanner). *Abbreviations:* ICV: intracranial volume.

Table S12: Follow-up analyses in patients for the effect of age at onset.

	Combined structures		Left hemisphere		Right hemisphere	
**Structure**	**Cohen's d**	**p-value**	**Cohen's d**	**p-value**	**Cohen's d**	**p-value**
ICV	0.01	0.9163				
Hippocampus	−0.07	0.5904	−0.16	0.2360	0.03	0.7992
Amygdala	−0.26	0.0628	−0.23	0.0935	−0.25	0.0705
Thalamus	−0.17	0.2196	−0.18	0.1955	−0.19	0.1562
Accumbens	−0.25	0.0702	−0.23	0.1012	−0.16	0.2581
Caudate	−0.16	0.2575	−0.08	0.5577	−0.22	0.1169
Putamen	0.10	0.4723	0.10	0.4757	0.10	0.4650
Pallidum	−0.11	0.4326	−0.19	0.1774	0	0.9852
Lateral Ventricle	0.18	0.1924	0.22	0.1206	0.17	0.2252

Notes Results of linear-mixed effect model within patients (fixed factors: AAO, age, sex and ICV; random factor: scanner). *Abbreviations:* AAO: age at onset, ICV: intracranial volume.

Table S13: Follow-up analyses in patients for the effect of duration of illness.

	**Combined structures**		**Left hemisphere**		**Right hemisphere**	
**Structure**	**Cohen's d**	**p-value**	**Cohen's d**	**p-value**	**Cohen's d**	**p-value**
ICV	−0.03	0.8386				
Hippocampus	0.05	0.6990	0.15	0.2612	−0.07	0.6326
Amygdala	0.25	0.0736	0.19	0.1600	0.24	0.0845
Thalamus	0.14	0.2949	0.13	0.3370	0.20	0.1518
Accumbens	0.26	0.0668	0.22	0.1209	0.18	0.2058
Caudate	0.15	0.2866	0.04	0.7674	0.24	0.0835
Putamen	−0.07	0.6177	−0.05	0.7273	−0.09	0.4960
Pallidum	0.14	0.2977	0.19	0.1782	0.07	0.6082
Lateral Ventricle	−0.17	0.2160	−0.22	0.1116	−0.15	0.2696

Notes: Results of linear-mixed effect model within patients (fixed factors: DOI, age, sex and ICV; random factor: scanner). *Abbreviations:* DOI: duration of illness, ICV: intracranial volume.

Table S14: Antipsychotic users/non-users compared to typically developing adolescents.

	**Combined structures**						**Left structures**						**Right structures**					
	**non-users**			**users**			**non-users**			**users**			**non-users**			**users**		
**Structure**	**d**	**t**	**p**	**d**	**t**	**p**	**d**	**t**	**p**	**d**	**t**	**p**	**d**	**t**	**p**	**d**	**t**	**p**
ICV	−0.15	−1.1	0.2720	−0.42	−4.79	**2e−06**												
Hippocampus	−0.30	−2.08	0.0378	−0.21	−2.37	0.0183	−0.25	−1.77	0.0779	−0.2	−2.25	0.0248	−0.25	−1.74	0.0824	−0.16	−1.77	0.0768
Amygdala	−0.22	−1.58	0.1157	−0.14	−1.63	0.1040	−0.18	−1.24	0.2142	−0.15	−1.69	0.0915	−0.20	−1.42	0.1550	−0.09	−1.06	0.2904
Thalamus	−0.06	−0.39	0.6940	−0.15	−1.69	0.0912	−0.04	−0.28	0.7792	−0.16	−1.79	0.0733	−0.07	−0.49	0.6242	−0.13	−1.47	0.1416
Accumbens	−0.10	−0.73	0.4656	−0.12	−1.33	0.1831	−0.21	−1.49	0.1373	−0.10	−1.09	0.2753	0.01	0.10	0.9203	−0.10	−1.09	0.2771
Caudate	0.14	1.00	0.3158	0.23	2.57	0.0104	0.17	1.20	0.2295	0.24	2.76	**0.0059**	0.10	0.70	0.4814	0.21	2.35	0.0190
Putamen	0.03	0.23	0.8162	0.17	1.94	0.0525	−0.05	−0.38	0.7064	0.18	2.00	0.0460	0.12	0.87	0.3874	0.14	1.61	0.1074
Pallidum	0.03	0.22	0.8230	0.22	2.52	0.0119	0.07	0.51	0.6069	0.26	2.98	**0.0031**	−0.02	−0.13	0.8957	0.14	1.64	0.1013
Lateral Ventricle	0.16	1.09	0.2769	0.22	2.42	0.0159	0.21	1.50	0.1342	0.22	2.50	0.0127	0.08	0.57	0.5682	0.22	2.43	0.0156

Notes: Results of linear-mixed effect model for antipsychotic medication effects (user/non-user) with controls (reference) (fixed factors: medication status, age, sex and ICV; random factor: scanner). *Abbreviations:* d: Cohen’s d, ICV: intracranial volume, p: p-value, t: t-value.

Table S15: Antipsychotic medication effects in patients only (reference antipsychotic medication non-users).

	**Combined structures**		**Left hemisphere**		**Right hemisphere**	
**Structure**	**Cohen's d**	**p-value**	**Cohen's d**	**p-value**	**Cohen's d**	**p-value**
ICV	−0.30	0.1044				
Hippocampus	0.19	0.3128	0.15	0.4288	0.18	0.3376
Amygdala	0.09	0.6339	0.01	0.9725	0.11	0.5609
Thalamus	−0.09	0.6349	−0.12	0.5227	−0.02	0.9051
Accumbens	−0.16	0.3943	0.06	0.7256	−0.33	0.0796
Caudate	−0.01	0.9656	−0.04	0.8398	0.05	0.7900
Putamen	0.09	0.6188	0.25	0.1735	−0.08	0.6728
Pallidum	0.24	0.1957	0.28	0.1325	0.15	0.4043
Lateral Ventricle	0.01	0.9410	−0.03	0.8722	0.08	0.6502

Notes: Results of linear-mixed effect model within patients for antipsychotic medication effects (fixed factors: medication user/non-user, age, sex and ICV; random factor: scanner). *Abbreviations:* DOI: duration of illness, ICV: intracranial volume.

Supplemental Method

Note S1: Image quality control

Visual quality control (QC) was performed at each site in order to exclude subjects where subcortical segmentation had failed. Each site computed a minimal list of segmentations to be checked for outliers based on subcortical volumes for each hemisphere in a given sample using a standardized script. Outliers were defined as being more than 2.698 standard deviations from the sample mean. For each subject in this minimal QC list, segmentations were visually inspected and excluded if segmentation errors had occurred.

Note S2: Meta analytical approach

To investigate heterogeneity between sites, we conducted an additional meta-analysis for the main model investigating patient-control differences, following a similar procedure as described in two previous ENIGMA publications [van Erp et al., 2016; Hibar et al., 2016] in R (version 3.5.2; www.r-project.org). Site-wise linear regression using the *lm*-function was conducted for each structure with diagnosis (patient-control status) as the variable of interest, while also adjusting for age, sex and intracranial volume (ICV) as a covariate. For sites with more than one scanner, this was included as a covariate. The Cohen's d effect sizes of patient-control differences and their standard errors were computed at each site [Nakagawa and Cuthill, 2007].

For each structure, we pooled the site-wise Cohen's d effect sizes and standard errors using an inverse variance-weighted random-effects model fitted by a restricted maximum-likelihood estimator, through the *rma*-function/*metafor* R package (version 2.0.0) [Viechtbauer, 2010]. This yields an estimate of the cross-site Cohen's d effect size, standard error and 95% confidence interval of the effect size, as well as the z- and p-values. Samples with less than 6 patients or controls were not included in the meta-analyses. Forest plots were generated using the *forest*-function from the *metafor*-package.

References

Chung Y, Addington J, Bearden CE, Cadenhead K, Cornblatt B, Mathalon DH, McGlashan T, Perkins D, Seidman LJ, Tsuang M, Walker E, Woods SW, McEwen S, Erp TGM van, Cannon TD (2018): Use of Machine Learning to Determine Deviance in Neuroanatomical Maturity Associated With Future Psychosis in Youths at Clinically High Risk. JAMA Psychiatry 75:960–968. https://jamanetwork.com/journals/jamapsychiatry/fullarticle/2686140.

Eckfeld A, Karlsgodt KH, Haut KM, Bachman P, Jalbrzikowski M, Zinberg J, van Erp TGM, Cannon TD, Bearden CE (2017): Disrupted Working Memory Circuitry in Adolescent Psychosis. Front Hum Neurosci 11. https://www.frontiersin.org/articles/10.3389/fnhum.2017.00394/full.

van Erp TG, Hibar DP, Rasmussen JM, Glahn DC, Pearlson GD, Andreassen OA, Agartz I, Westlye LT, Haukvik UK, Dale AM, Melle I, Hartberg CB, Gruber O, Kraemer B, Zilles D, Donohoe G, Kelly S, McDonald C, Morris DW, Cannon DM, Corvin A, Machielsen MW, Koenders L, de Haan L, Veltman DJ, Satterthwaite TD, Wolf DH, Gur RC, Gur RE, Potkin SG, Mathalon DH, Mueller BA, Preda A, Macciardi F, Ehrlich S, Walton E, Hass J, Calhoun VD, Bockholt HJ, Sponheim SR, Shoemaker JM, van Haren NE, Pol HE, Ophoff RA, Kahn RS, Roiz-Santianez R, Crespo-Facorro B, Wang L, Alpert KI, Jonsson EG, Dimitrova R, Bois C, Whalley HC, McIntosh AM, Lawrie SM, Hashimoto R, Thompson PM, Turner JA (2016): Subcortical brain volume abnormalities in 2,028 individuals with schizophrenia and 2,540 healthy controls via the ENIGMA consortium. Mol Psychiatry 21:585.

Gurholt TP, Osnes K, Nerhus M, Jørgensen KN, Lonning V, Berg AO, Andreassen OA, Melle I, Agartz I (2018): Vitamin D, Folate and the Intracranial Volume in Schizophrenia and Bipolar Disorder and Healthy Controls. Sci Rep 8:10817.

Hibar DP, Westlye LT, van Erp TG, Rasmussen J, Leonardo CD, Faskowitz J, Haukvik UK, Hartberg CB, Doan NT, Agartz I, Dale AM, Gruber O, Kramer B, Trost S, Liberg B, Abe C, Ekman CJ, Ingvar M, Landen M, Fears SC, Freimer NB, Bearden CE, Costa Rica/Colombia Consortium for Genetic Investigation of Bipolar E, Sprooten E, Glahn DC, Pearlson GD, Emsell L, Kenney J, Scanlon C, McDonald C, Cannon DM, Almeida J, Versace A, Caseras X, Lawrence NS, Phillips ML, Dima D, Delvecchio G, Frangou S, Satterthwaite TD, Wolf D, Houenou J, Henry C, Malt UF, Boen E, Elvsashagen T, Young AH, Lloyd AJ, Goodwin GM, Mackay CE, Bourne C, Bilderbeck A, Abramovic L, Boks MP, van Haren NE, Ophoff RA, Kahn RS, Bauer M, Pfennig A, Alda M, Hajek T, Mwangi B, Soares JC, Nickson T, Dimitrova R, Sussmann JE, Hagenaars S, Whalley HC, McIntosh AM, Thompson PM, Andreassen OA (2016): Subcortical volumetric abnormalities in bipolar disorder. Mol Psychiatry 21:1710–1716.

Juuhl-Langseth M, Rimol LM, Rasmussen Jr IA, Thormodsen R, Holmén A, Emblem KE, Due-Tønnessen P, Rund BR, Agartz I (2012): Comprehensive segmentation of subcortical brain volumes in early onset schizophrenia reveals limited structural abnormalities. Psychiatry Res Neuroimaging 203:14–23.

Nakagawa S, Cuthill IC (2007): Effect size, confidence interval and statistical significance: a practical guide for biologists. Biol Rev Camb Philos Soc 82:591–605.

Tesli N, van der Meer D, Rokicki J, Storvestre G, Røsæg C, Jensen A, Hjell G, Bell C, Fischer-Vieler T, Tesli M, Andreassen OA, Melle I, Agartz I, Haukvik UK (2020): Hippocampal subfield and amygdala nuclei volumes in schizophrenia patients with a history of violence. Eur Arch Psychiatry Clin Neurosci.

Viechtbauer W (2010): Conducting Meta-Analyses in R with the metafor Package. J Stat Softw 36:1–48.

Wedervang-Resell K, Friis S, Lonning V, Smelror RE, Johannessen C, Agartz I, Ulven SM, Holven KB, Andreassen OA, Myhre AM (2019): Lipid alterations in adolescents with early-onset psychosis may be independent of antipsychotic medication. Schizophr Res. http://www.sciencedirect.com/science/article/pii/S0920996419305468.
